# Real-world comparative effectiveness of first-line abemaciclib versus palbociclib in HR+/HER2- metastatic breast cancer: A propensity-matched retrospective analysis

**DOI:** 10.1016/j.breast.2025.104597

**Published:** 2025-10-12

**Authors:** Cho-Hao Lee, Po-Huang Chen, Hong-Jie Jhou, Wei-Cheng Chang, Hsin-Yu Chen, Li-Ting Kao, Tina Yi-Jin Hsieh, Ming-Shen Dai

**Affiliations:** a Division of Hematology and Oncology, Department of Internal Medicine, Tri-Service General Hospital, National Defense Medical University, Taipei, Taiwan; b Department of Oncology, Tri-Service General Hospital, National Defense Medical University, Taipei, Taiwan; c Neurological Institute, Changhua Christian Hospital, Changhua, Taiwan; d Department of Ophthalmology, Taoyuan Hospital, Ministry of Health and Welfare, Taiwan; e Department of Family Medicine, Chi Mei Medical Center, Tainan, Taiwan; f Graduate Institute of Life Sciences, National Defense Medical University, Taipei, Taiwan; g School of Pharmacy, National Defense Medical University, Taipei, Taiwan; h Department of Pharmacy Practice, Tri-Service General Hospital, Taipei, Taiwan; i Department of Obstetrics & Gynecology, Beth Israel Deaconess Medical Center, Boston, MA, USA

**Keywords:** Metastatic breast cancer, CDK4/6 inhibitors, Hormone receptor-positive, Target trial emulation, Real-world data

## Abstract

**Background:**

In the first-line treatment of hormone receptor-positive, HER2-negative (HR+/HER2-) metastatic breast cancer (mBC), the comparative effectiveness of different cyclin-dependent kinase 4/6 inhibitors (CDK4/6i) remains unclear due to the absence of head-to-head randomized trials. We aimed to compare the real-world outcomes of abemaciclib versus palbociclib.

**Methods:**

We performed a retrospective, propensity-matched cohort study using the TriNetX Analytics Network database (2014–2025). The primary outcome was overall survival (OS). To ensure robust findings, the analysis was supported by multiple sensitivity tests, including restricted mean survival time (RMST) to provide a model-free effect measure, and E-value analysis to quantify the potential impact of unmeasured confounding.

**Results:**

From 15,830 eligible patients, we created a matched cohort of 2768 patients on abemaciclib and 2768 on palbociclib. After a median follow-up of 33.7 months for the abemaciclib group and 44.2 months for the palbociclib group, treatment with abemaciclib was associated with significantly longer median OS (6.0 vs. 5.0 years; HR 0.80, 95 % CI 0.72–0.90; p < 0.001). The RMST analysis confirmed a significant survival benefit of 5.96 months over the follow-up period (p < 0.001). Abemaciclib was associated with lower rates of neutropenia but higher rates of diarrhea. The survival advantage was consistent across sensitivity and subgroup analyses.

**Conclusions:**

In this large, real-world cohort study, first-line abemaciclib was associated with a significant overall survival benefit compared to palbociclib for patients with HR+/HER2-mBC. This finding was robust across multiple sensitivity analyses. These results provide valuable evidence to inform treatment decisions in the absence of direct randomized trial data.

## Highlights

• Abemaciclib showed superior OS vs palbociclib (median 6.0 vs 5.0 years)
• Lower composite hematologic toxicity with abemaciclib (39.2 % vs 54.2 %)
• Higher diarrhea with abemaciclib (24.6 % vs 19.7 %), hospitalizations not increased
• Survival benefit confirmed in on-treatment and landmark sensitivity analyses

## 1 Introduction

Cyclin-dependent kinase 4/6 inhibitors (CDK4/6i) in combination with endocrine therapy have fundamentally transformed the treatment landscape of hormone receptor-positive, HER2-negative (HR+/HER2-) metastatic breast cancer (mBC). Landmark randomized trials have consistently demonstrated that adding a CDK4/6i (palbociclib, ribociclib, or abemaciclib) to first-line endocrine therapy significantly improves progression-free survival by 10–15 months compared to endocrine therapy alone, with overall survival benefits ranging from 12.9 to 28.8 months depending on the agent and clinical setting [1,2]. Currently, over 80 % of patients with HR+/HER2-metastatic breast cancer receive CDK4/6 inhibitors as first-line therapy globally, making the choice between these agents one of the most consequential treatment decisions in modern breast oncology [3]. However, the optimal selection among the three available CDK4/6 inhibitors remains a critical clinical challenge, as no head-to-head randomized trials have been conducted to directly compare their relative efficacy and safety profiles [4].

Abemaciclib and palbociclib, two of the most widely prescribed CDK4/6 inhibitors, exhibit distinct pharmacologic and clinical characteristics that may translate into differential patient outcomes. Abemaciclib is administered continuously without scheduled treatment breaks and demonstrates more selective CDK4 inhibition, along with activity against additional kinases including CDK9, potentially contributing to enhanced anti-tumor efficacy [5]. Conversely, palbociclib follows an intermittent dosing schedule (21 days on, 7 days off) with more balanced CDK4/6 inhibition. These mechanistic differences manifest in contrasting safety profiles: abemaciclib is associated with higher rates of gastrointestinal toxicity, particularly diarrhea (80–90 % any grade), while palbociclib more frequently causes dose-limiting neutropenia (75–80 % any grade) requiring treatment interruptions [6]. Additionally, abemaciclib's ability to cross the blood-brain barrier and maintain continuous target engagement may confer unique therapeutic advantages in certain patient populations.

Despite the clinical importance of treatment selection, comparative effectiveness data remain limited and contradictory. Network meta-analyses have yielded conflicting results, with some suggesting equivalent efficacy among CDK4/6 inhibitors [7,8], while others indicate potential superiority of specific agents in certain endpoints or patient subgroups [9,10]. Real-world evidence has been similarly inconsistent. The recently published large-scale Flatiron Health analysis by Rugo et al. (2025) found no significant overall survival differences between abemaciclib and palbociclib when combined with aromatase inhibitors (adjusted HR 0.95, 95 % CI 0.84–1.08) [11]. However, several institutional studies have suggested potential advantages for abemaciclib, particularly in progression-free survival and in endocrine-sensitive populations [12,13]. These discrepancies likely reflect differences in study populations, analytical methodologies, sample sizes, and potential residual confounding, highlighting the need for larger, more rigorously designed comparative studies.

Emerging evidence suggests that abemaciclib's unique pharmacokinetic and pharmacodynamic properties may translate into clinically meaningful benefits that have not been fully captured in previous analyses [14]. Beyond its continuous dosing and brain penetration, abemaciclib is the only CDK4/6 inhibitor with demonstrated single-agent activity in heavily pretreated breast cancer and proven overall survival benefit in the adjuvant setting (monarchE trial) [15]. Its distinct mechanism of action, including inhibition of CDK9 and subsequent effects on transcriptional regulation, may contribute to more durable responses and improved long-term outcomes [16]. Furthermore, the lower incidence of neutropenia-related dose modifications with abemaciclib may allow for more consistent drug delivery and optimal therapeutic exposure in real-world settings [17].

To address these critical knowledge gaps and provide definitive comparative effectiveness evidence, we conducted one of the largest real-world retrospective studies to date comparing first-line abemaciclib versus palbociclib in HR+/HER2-metastatic breast cancer. Leveraging the TriNetX Analytics Network database encompassing over 15,000 patients, we employed rigorous target trial emulation methodology with propensity score matching to minimize confounding and approximate the conditions of a randomized clinical trial. Our primary objective was to evaluate overall survival differences between these agents, while comprehensively assessing their comparative safety profiles and identifying patient subgroups who may derive greater benefit from specific treatments. We hypothesized that abemaciclib's more continuous CDK4/6 inhibition and distinct mechanism of action would translate into improved long-term survival outcomes compared to palbociclib, despite the inherent limitations of observational data.

## 2 Methods

### 2.1 Study design and data source

We conducted a retrospective cohort study designed as a target trial emulation comparing first-line endocrine therapy plus either abemaciclib or palbociclib for HR+/HER2-metastatic breast cancer (eTable 1). De-identified patient data were obtained from the TriNetX Analytics Network, a global federated network that aggregates longitudinal clinical data from over 100 healthcare organizations, primarily in the United States, Europe, and Asia. The participating institutions include a diverse mix of academic medical centers, specialty clinics, and community hospitals (eTable 2). The study period spanned January 1, 2014 through March 1, 2025, with March 1, 2025 serving as the administrative censoring date (end of follow-up). The study was approved by the institutional review board of the participating institutions as required, with a waiver of informed consent for use of de-identified data.

### 2.2 Population and eligibility criteria

The cohort included adult patients (≥18 years) with a diagnosis of HR+/HER2-metastatic breast cancer who initiated first-line systemic therapy with either abemaciclib or palbociclib in combination with endocrine therapy. Metastatic disease was defined by documentation of breast cancer with distant metastatic sites. We required that the start of abemaciclib or palbociclib (with concurrent endocrine therapy) was the first systemic treatment for metastatic disease (new-user design).

HER2-negative status was confirmed by: (1) immunohistochemistry (IHC) results indicating a score of 0 or 1+, or a score of 2+ with negative fluorescence *in situ* hybridization (FISH) testing, or (2) absence of HER2-targeted therapy use (e.g., trastuzumab, pertuzumab, ado-trastuzumab emtansine, trastuzumab deruxtecan, neratinib, lapatinib, tucatinib, or margetuximab) in patients receiving hormonal therapy for metastatic breast cancer.

To ensure a new-user cohort, we excluded patients who had any chemotherapy, targeted therapy, or CDK4/6 inhibitor before the initiation of the study drugs in the metastatic setting. Patients who received a CDK4/6 inhibitor in an adjuvant trial or any investigational systemic therapy prior to or concomitant with first-line treatment were also excluded. Additionally, prior use of any CDK4/6 inhibitor (in any setting) was an exclusion, to focus on first exposure; and we excluded patients who used CDK4/6 inhibitor without concurrent endocrine therapy. Furthermore, anti-cancer treatment should start within 6 months after metastatic breast cancer diagnosis.

We applied further criteria to emulate a trial-like population (eTable 1). Patients with an Eastern Cooperative Oncology Group (ECOG) performance status of 3–4 (or equivalent poor functional status) at baseline were excluded, as were those with evidence of end-stage organ dysfunction likely to impact short-term survival (e.g., uncontrolled severe comorbid conditions within 3 months prior to therapy initiation). These exclusions were implemented to reduce immortal time bias and ensure that patients in both groups were fit to receive therapy. Eligible patients were classified into two groups based on the first CDK4/6 inhibitor received in the metastatic setting: abemaciclib or palbociclib. The date of first drug administration (combined with endocrine therapy) was defined as the index date for time-to-event analyses (median time from diagnosis to treatment 59 days in abemaciclib versus 58 days in palbociclib, eTable 10).

### 2.3 Treatment exposure

The exposures of interest were first-line abemaciclib plus endocrine therapy versus palbociclib plus endocrine therapy. For both groups, the choice of endocrine partner (aromatase inhibitor vs fulvestrant vs tamoxifen) was at the treating physician's discretion and were recorded from medication administration data. Patients remained in their initial exposure group for as-started (equivalent to intention-to-treat, at least two prescription records) analyses regardless of dose reductions, temporary interruptions, or subsequent treatment changes (for survival, patients were followed until death or end of study even if therapies changed). Mean CDK4/6i treatment episodes were 7.1 ± 9.6 times in abemaciclib versus 7.7 ± 11.6 in palbociclib (eTable 10).

### 2.4 Outcomes and measures

The primary outcome was overall survival (OS), defined as the time from initiation of abemaciclib or palbociclib (index date) to death from any cause. Patients without a recorded death were censored at the date of last known clinical encounter or end of the study period (March 1, 2025), whichever came first. Death information was obtained from a combination of EHR documentation and linked mortality records when available.

Key secondary outcomes were chosen to assess the comparative safety and effectiveness-related events of the two treatments, using a combination of EHR diagnosis codes, procedure codes, and clinical measurements. These secondary endpoints included.

• **All-cause hospitalization**: any inpatient hospital admission occurring after treatment initiation, as identified by hospital admission records. We also examined ICU admission (a subset of hospitalizations involving intensive care unit level care) as a marker of severe acute events.
• **Severe infection**: defined as any infection requiring hospitalization or meeting sepsis criteria, including febrile neutropenia or opportunistic infections. These were identified via ICD-10 diagnosis codes for sepsis, neutropenic fever, pneumonia, etc.
• **Hematologic adverse events**: including neutropenia, leukopenia, anemia, and thrombocytopenia. We captured these events primarily via diagnostic codes (ICD-10 codes for neutropenia, pancytopenia, anemia, etc.). We also evaluated a composite hematologic toxicity outcome indicating the occurrence of any of the above blood count abnormalities.
• **Non-hematologic adverse events**: we focused on those known to differ between the drugs, including:
  o Gastrointestinal toxicity (primarily diarrhea, but also nausea/vomiting, stomatitis, and decreased appetite)
  o Cardiac events (including arrhythmias like atrial fibrillation, ischemic heart disease, pericardial disease, hypertension, heart failure, and QT interval prolongation)
  o Dermatologic toxicity (alopecia, rash, and stomatitis)
  o Others: hepatic toxicity (elevated liver enzymes or drug-induced liver injury codes), fatigue, headache, pyrexia, interstitial lung disease and parageusia.

These events were identified through ICD-10 codes and, for selected labs (e.g., liver enzymes), through lab results if available (eTable 2). All outcomes were assessed from the time of treatment initiation until an event or censoring. For adverse events, patients were censored at last encounter if still on drug at end of follow-up.

The “on-treatment” (also called per-protocol) approach is defined by using abemaciclib or palbociclib for at least 6 consecutive incidences to confirm its continuity. Overall survival was analyzed in an as-started manner (patients were followed for survival regardless of treatment changes, to capture the overall effectiveness of the initial strategy).

### 2.5 Propensity score matching

To control for confounding due to non-random treatment assignment, we performed propensity score matching between the abemaciclib and palbociclib groups. A propensity score for receiving abemaciclib (versus palbociclib) was estimated for each patient using a multivariable logistic regression model.

Baseline covariates in the propensity model included: age at treatment initiation (as a continuous variable), sex, race/ethnicity, ECOG performance status, body mass index (BMI), key comorbidities (including presence of diabetes, hypertension, coronary artery disease, chronic lung disease, liver disease, chronic kidney disease, cerebral infarction, liver cirrhosis, systemic lupus erythematosus, and psoriasis), social determinants of health (problems related to social environment, housing/economic circumstances, employment, and education/literacy), obesity status, choice of endocrine partner (aromatase inhibitor vs fulvestrant vs tamoxifen) for the first-line regimen, and individual metastatic sites (brain, bone, lung, liver, and peritoneal metastases as separate binary variables).

Due to TriNetX platform constraints, composite measures of disease burden (such as total number of metastatic sites ≥2 or visceral metastasis status) could not be directly incorporated into the propensity score model but were evaluated in pre-specified subgroup analyses. These variables were selected a priori based on clinical relevance and data availability, aiming to approximate the conditions of a randomized trial.

Patients in the abemaciclib group were matched 1:1 to patients in the palbociclib group using nearest-neighbor matching on the logit of the propensity score, with a caliper of 0.2 standard deviations of the score and without replacement. The matching was performed at the time of analysis and was not time-dependent (i.e., baseline matching). Balance between the matched groups was assessed by standardized mean differences (SMD) for each covariate; an SMD <0.1 was considered indicative of good balance.

After matching, covariate distributions were compared to ensure no residual substantial imbalances. The primary analyses of outcomes were conducted on this propensity-matched cohort, which emulates a randomized population of patients receiving abemaciclib vs palbociclib with similar baseline characteristics.

### 2.6 Statistical analysis

We summarized baseline characteristics using means (standard deviations) for continuous variables and frequencies (percentages) for categorical variables. Matched group comparisons used paired statistical tests as appropriate (e.g., paired *t*-test for continuous measures, McNemar's test for binary measures) purely to confirm balance; however, emphasis was placed on SMD rather than p-values for baseline differences due to the large sample size.

For the primary outcome of overall survival, we plotted Kaplan-Meier survival curves for each treatment group in the matched sample and used the log-rank test to compare survival distributions. Cox proportional hazards models were used to estimate the hazard ratio (HR) for death for abemaciclib vs palbociclib, with robust standard errors to account for matching. The proportional hazards assumption was checked via Schoenfeld residuals and by examining log-log survival plots; no significant violations were detected. We also calculated median survival times with 95 % confidence intervals using the Kaplan-Meier method (eTable 11).

We conducted a restricted mean survival time (RMST) analysis to complement Cox regression, providing a robust, clinically interpretable measure of treatment effect without relying on the proportional hazards assumption. RMST, the average survival time up to τ = 4624 days (151.9 months, the minimum of maximum follow-up times across groups), was calculated as the area under the Kaplan-Meier curve using numerical integration (trapezoidal rule). Standard errors were derived from Kaplan-Meier survival probabilities and their 95 % confidence intervals, with differences between groups tested via a two-sample Z-test. This analysis supports the reliability of our Kaplan-Meier survival curves, especially when proportional hazards may not hold.

Secondary outcomes were analyzed with cumulative incidence functions and hazard or risk ratios as appropriate. For binary safety endpoints that could occur at any time on treatment, we calculated the proportion of patients experiencing the event in each group by certain time landmarks (e.g., at 1 month, 3 months, 6 months, 1 year, 3 years, 5 years, and by end of follow-up) and computed risk ratios (RRs) with 95 % CIs. These RRs were derived from Kaplan-Meier estimates at specific time points from cumulative incidence in the matched groups.

Risk ratios were calculated at pre-specified time points (1, 3, 6 months, 1, 3, and 5 years) and overall (end of follow-up) using Kaplan-Meier estimates of cumulative incidence. The overall risk ratio represents the ratio of cumulative risks at the end of follow-up. We acknowledge that hazard ratios and risk ratios may differ in direction or magnitude when the proportional hazards assumption is violated or when there are time-varying treatment effects. Both measures are presented to provide complementary perspectives: HRs reflect the average hazard over the entire follow-up, while time-specific RRs capture the cumulative risk at distinct clinical timepoints. In addition, we reported the overall relative risk over the entire follow-up (which is mathematically related to the area-under-curve of the cumulative incidence). Subgroup analyses of overall survival were pre-specified for key strata: age (18–65 vs ≥ 65 years), race (White, Black or Asian), gender (female or male), menopausal status (pre or post), presence of brain metastasis, type of endocrine partner (aromatase inhibitor vs fulvestrant), ≥2 metastatic sites, and presence of visceral metastasis. For each subgroup, we estimated the treatment HR for OS and assessed interaction terms in the Cox model to test for heterogeneity of the treatment effect across subgroups. We present subgroup HRs with confidence intervals and p-values for interaction; these analyses were considered exploratory.

To assess the robustness of our findings to potential unmeasured confounding, we calculated E-values for statistically significant associations. The E-value represents the minimum strength of association that an unmeasured confounder would need to have with both the treatment and outcome to fully explain away the observed effect, conditional on measured covariates. E-values were calculated for the point estimate of the hazard ratio using established methods. Higher E-values indicate greater robustness to unmeasured confounding.

Sensitivity analyses were performed to evaluate the robustness of the findings, including: (1) an on-treatment analysis (per-protocol, requiring at least 6 administrations); (2) landmark analysis (exclusion of events occurring within 3 months after the index date); (3) a calendar time-restricted analysis, in which both experimental and control cohorts were limited to patients diagnosed in 2019 or later, to account for potential improvements in subsequent-line therapies that may influence overall survival.

Results from these analyses were qualitatively compared with the primary findings. Negative control comparisons used non-relevant outcomes like kidney stones, gallbladder stones and burns as negative controls to validate our study design.

All statistical tests were two-sided, with p < 0.05 indicating statistical significance. No adjustment was made for multiple comparisons in secondary endpoints, so those results should be interpreted as exploratory. Analyses were performed using R version 4.2 and TriNetX Analytics platform.

## 3 Results

### 3.1 Patient characteristics

**Fig. 1:**
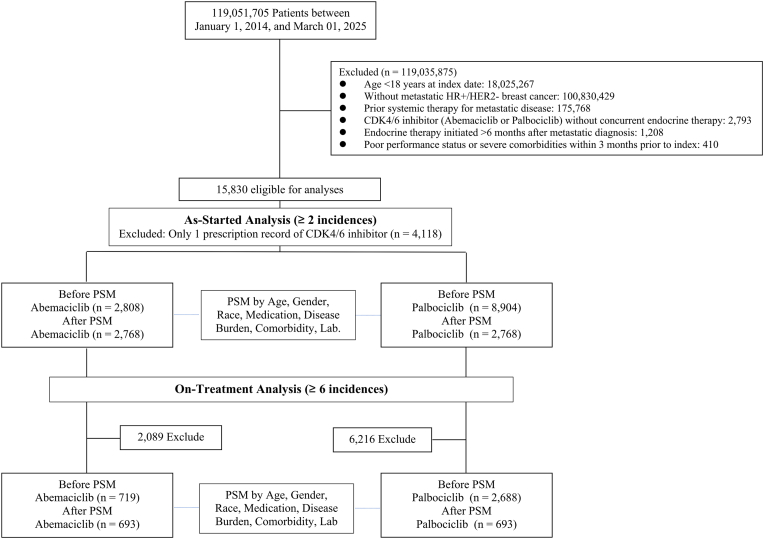
Cohort Selection Flow Diagram CONSORT-style flowchart depicting the systematic patient selection process and study design. The diagram shows patient filtering through inclusion and exclusion criteria, division into treatment cohorts, and propensity score matching procedures.

A total of 15,830 patients met inclusion criteria, with 2808 receiving abemaciclib and 8904 receiving palbociclib, while 4118 patients were excluded due to only one incidence of CDK4/6 inhibitor (Fig. 1). Prior to matching, notable differences existed: abemaciclib patients were younger (mean age 61.2 vs 64.5 years), had different endocrine partner distributions (more anastrozole/tamoxifen vs letrozole/fulvestrant), higher obesity prevalence (20.4 % vs 14.3 %), and more brain metastases (7.8 % vs 6.0 %) with fewer bone, lung, and liver metastases (Table 1).

**Table 1:** Baseline Characteristics Before and After Propensity Score Matching

Characteristics	Before Propensity Score Matching				After Propensity Score Matching			
	Abemaciclib (n=2,808)	Palbociclib (n=8,904)	P Value	SMD	Abemaciclib (n=2,768)	Palbociclib (n=2,768)	P Value	SMD
**Demographics**								
Age at index, mean (SD), y	61.2 (13.4)	64.5 (12.6)	<.001	0.252	61.4 (13.4)	61.8 (13.2)	0.455	0.031
**Sex, No. (%)**								
Female	2,669 (95.0)	8,417 (94.5)	0.12	0.023	2,629 (95.0)	2,634 (95.2)	0.207	0.008
Male	33 (1.2)	120 (1.3)	0.411	0.015	33 (1.2)	32 (1.2)	0.058	0.003
Unknown	106 (3.8)	367 (4.1)	0.392	0.017	106 (3.8)	102 (3.7)	0.808	0.008
**Race/Ethnicity, No. (%)**								
White	1,891 (67.3)	6,384 (71.7)	<.001	0.095	1,875 (67.7)	1,897 (68.5)	0.533	0.017
Black or African American	364 (13.0)	1,110 (12.5)	0.392	0.015	359 (13.0)	352 (12.7)	0.97	0.008
Asian	173 (6.2)	293 (3.3)	<.001	0.136	157 (5.7)	153 (5.5)	0.615	0.006
Unknown	249 (8.9)	735 (8.3)	0.28	0.022	249 (9.0)	242 (8.7)	0.492	0.009
**Comorbidities, No. (%)**								
Diabetes mellitus	462 (16.5)	1,420 (15.9)	0.739	0.014	456 (16.5)	440 (15.9)	0.506	0.016
Hypertensive diseases	1,121 (39.9)	3,653 (41.0)	0.163	0.023	1,109 (40.1)	1,116 (40.3)	0.879	0.005
Ischemic heart diseases	279 (9.9)	845 (9.5)	0.535	0.015	273 (9.9)	269 (9.7)	0.618	0.005
Heart failure	171 (6.1)	552 (6.2)	0.824	0.005	168 (6.1)	156 (5.6)	0.808	0.018
Chronic obstructive pulmonary disease	126 (4.5)	470 (5.3)	0.051	0.037	126 (4.6)	118 (4.3)	0.953	0.014
Chronic kidney disease	329 (11.7)	1,051 (11.8)	0.558	0.003	326 (11.8)	306 (11.1)	0.758	0.023
Cerebral infarction	64 (2.3)	167 (1.9)	0.316	0.028	63 (2.3)	59 (2.1)	0.631	0.01
Cirrhosis of liver	33 (1.2)	93 (1.0)	0.097	0.012	32 (1.2)	36 (1.3)	0.249	0.013
Systemic lupus erythematosus	20 (0.7)	29 (0.3)	0.014	0.054	17 (0.6)	16 (0.6)	0.204	0.005
Psoriasis	28 (1.0)	92 (1.0)	0.528	0.004	26 (0.9)	23 (0.8)	0.36	0.012
Overweight/obesity	573 (20.4)	1,272 (14.3)	<.001	0.162	555 (20.1)	523 (18.9)	0.823	0.029
**Metastatic Sites, No. (%)**								
Bone metastases	1,091 (38.8)	4,984 (56.0)	<.001	0.349	1,078 (38.9)	1,100 (39.7)	0.479	0.017
Brain metastases	220 (7.8)	538 (6.0)	0.002	0.071	217 (7.8)	215 (7.8)	0.587	0.003
Lung metastases	264 (9.4)	1,304 (14.6)	<.001	0.162	260 (9.4)	280 (10.1)	0.309	0.025
Liver metastases	296 (10.5)	1,161 (13.0)	<.001	0.078	285 (10.3)	299 (10.8)	0.479	0.016
Peritoneal metastases	45 (1.6)	212 (2.4)	0.002	0.056	45 (1.6)	46 (1.7)	0.85	0.004
**Concomitant Endocrine Therapy, No. (%)**								
Tamoxifen	286 (10.2 %)	766 (8.6 %)	0.011	0.054	279 (10.1 %)	274 (9.9 %)	0.823	0.006
Fulvestrant	374 (13.3 %)	1,616 (18.1 %)	<.001	0.133	374 (13.5 %)	377 (13.6 %)	0.906	0.003
Exemestane	281 (10.0 %)	827 (9.3 %)	0.256	0.024	280 (10.1 %)	266 (9.6 %)	0.528	0.017
Letrozole	923 (32.9 %)	3,798 (42.7 %)	<.001	0.203	919 (33.2 %)	895 (32.3 %)	0.492	0.018
Anastrozole	944 (33.6 %)	1,897 (21.3 %)	<.001	0.279	917 (33.1 %)	956 (34.5 %)	0.268	0.03
**Clinical Measurements**								
ECOG performance status, mean (SD)	0.5 (0.6)	0.8 (1.0)	0.069	0.361	0.4 (0.6)	0.5 (0.9)	0.263	0.075
Body mass index, mean (SD), kg/m^2^	29.2 (7.1)	29.0 (7.2)	0.554	0.025	29.1 (7.1)	29.5 (7.4)	0.106	0.045
**Social Determinants of Health, No. (%)**								
Problems related to social environment	19 (0.7)	34 (0.4)	0.009	0.041	16 (0.6)	12 (0.4)	0.63	0.02
Problems related to housing/economic circumstances	51 (1.8)	77 (0.9)	<.001	0.083	44 (1.6)	41 (1.5)	0.921	0.009
Problems related to employment	16 (0.6)	10 (0.1)	<.001	0.079	10 (0.4)	10 (0.4)	>.999	<0.001
Problems related to education/literacy	10 (0.4)	10 (0.1)	0.017	0.05	10 (0.4)	10 (0.4)	>.999	<0.001

Abbreviations: BMI, body mass index; ECOG, Eastern Cooperative Oncology Group; SD, standard deviation; SMD, standardized mean difference.

Note: A standardized mean difference (SMD) <0.10 indicates good balance between groups after propensity score matching. P values were calculated using chi-square tests for categorical variables and t-tests for continuous variables. Percentages may not sum to 100 % due to rounding. The matched cohorts were derived using 1:1 nearest-neighbor propensity score matching with a caliper of 0.2 standard deviations of the logit of the propensity score. Totals for concomitant endocrine therapy may exceed 100 % as patients could switch or receive more than one endocrine partner during the follow-up period.

After 1:1 propensity score matching, 2768 patients remained in each group (N = 5536), with median follow-up of 33.7 months (1027 days) in the abemaciclib group versus 44.2 months (1347 days) in the palbociclib group (eTable 11). Baseline characteristics were well-balanced with mean age ∼61.6 years, 95 % female, and similar racial composition (∼68 % White, 13 % Black, 6 % Asian). Comorbidity prevalence was nearly identical: diabetes (∼16 %), cardiovascular disease (∼10 %). Endocrine therapy distribution was balanced with ∼78 % receiving aromatase inhibitors and ∼24 % non-aromatase inhibitors (14 % fulvestrant). All standardized mean differences were <0.1, indicating successful matching (Table 1). The matched cohort therefore represented two similar patient groups differing mainly in whether abemaciclib or palbociclib was used in their initial treatment.

### 3.2 Overall survival (Fig. 2)

**Fig. 2:**
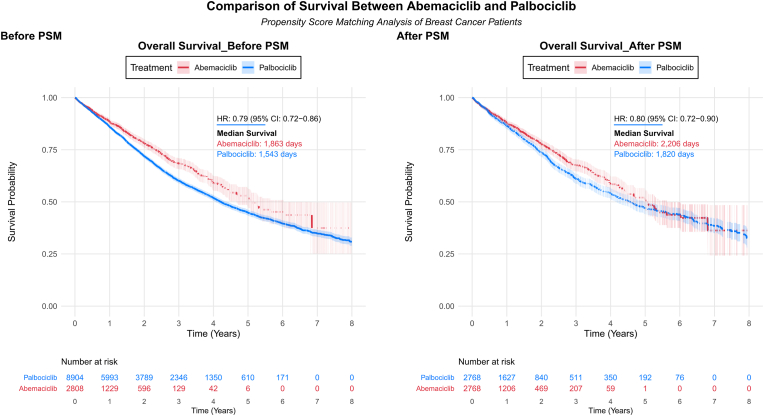
Overall Survival of Patients Treated with Abemaciclib vs. Palbociclib Kaplan-Meier survival curves comparing treatment outcomes before (left panel) and after (right panel) propensity score matching. Treatment groups are distinguished by color (red: abemaciclib, blue: palbociclib), with shaded areas representing 95 % confidence intervals. Numbers at risk tables below each plot indicate patient follow-up at specified time intervals in years. (For interpretation of the references to color in this figure legend, the reader is referred to the Web version of this article.)

In the propensity-matched cohort, abemaciclib demonstrated significantly longer overall survival compared to palbociclib (Fig. 2). Median OS was 6.0 years (95 % CI, 5.6–6.5) with abemaciclib versus 5.0 years (95 % CI, 4.6–5.2) with palbociclib. The hazard ratio for death was 0.80 (95 % CI 0.72–0.90; P < 0.001), representing a 20 % reduction in death risk. The E-value for this association was 1.81, indicating that an unmeasured confounder would need to be associated with both treatment selection and mortality by a risk ratio of 1.81-fold each, above and beyond the measured confounders, to fully explain away the observed survival benefit. Survival curves separated within the first year and maintained separation throughout follow-up. By study end, survival probability was 42.8 % with abemaciclib versus 29.3 % with palbociclib.

Complementary RMST analysis corroborated the survival advantage of abemaciclib (Table 2, eFig. 1). Within the restriction time of 151.9 months, patients treated with abemaciclib had a mean survival time of 79.17 months (95 % CI, 75.99–82.36) compared to 73.21 months (95 % CI, 71.84–74.59) with palbociclib. The difference in RMST was 5.96 months (95 % CI, 2.49–9.43; P < 0.001), representing the average gain in survival time attributable to abemaciclib. This translates to approximately 6 additional months of life expectancy over the follow-up period, providing a clinically meaningful and readily interpretable measure of the treatment benefit that complements the hazard ratio analysis.

**Table 2:** Key Clinical Outcomes and Adverse Events Comparing Abemaciclib versus Palbociclib

Outcome	Hazard Ratio (95 % CI)	Risk Ratio at 1 Year (95 % CI)	Risk Ratio at 3 Years (95 % CI)	Overall Risk Ratio (95 % CI)
**PRIMARY OUTCOME**				
Overall Survival	0.80 (0.72, 0.90)∗	0.90 (0.73, 1.11)	0.78 (0.66, 0.93)∗	0.55 (0.51, 0.60)∗
**CLINICAL EVENTS**				
Hospitalization	0.93 (0.81, 1.06)	1.13 (0.83, 1.53)	0.93 (0.70, 1.23)	0.68 (0.61, 0.77)∗
ICU Admission	1.00 (0.85, 1.16)	1.11 (0.86, 1.43)	1.33 (1.05, 1.69)∗	0.69 (0.60, 0.79)∗
Severe Infections	0.85 (0.75, 0.96)∗	0.90 (0.75, 1.09)	0.82 (0.69, 0.97)∗	0.63 (0.56, 0.70)∗
**HEMATOLOGIC ADVERSE EVENTS**				
Neutropenia	0.52 (0.47, 0.59)∗	0.53 (0.46, 0.62)∗	0.51 (0.44, 0.58)∗	0.48 (0.44, 0.54)∗
Leukopenia	0.63 (0.52, 0.76)∗	0.57 (0.43, 0.76)∗	0.57 (0.44, 0.74)∗	0.52 (0.43, 0.63)∗
Anemia	1.08 (0.98, 1.18)	1.07 (0.95, 1.21)	1.07 (0.96, 1.19)	0.84 (0.78, 0.90)∗
Thrombocytopenia	0.97 (0.84, 1.12)	1.01 (0.81, 1.25)	1.05 (0.85, 1.29)	0.73 (0.64, 0.83)∗
Hematologic Toxicity (Composite)	0.79 (0.73, 0.86)∗	0.80 (0.73, 0.87)∗	0.78 (0.72, 0.85)∗	0.72 (0.68, 0.77)∗
**GASTROINTESTINAL ADVERSE EVENTS**				
Diarrhea	1.83 (1.63, 2.05)∗	2.52 (2.13, 2.99)∗	2.10 (1.80, 2.44)∗	1.25 (1.13, 1.38)∗
Nausea & Vomiting	0.95 (0.86, 1.04)	1.03 (0.92, 1.16)	0.90 (0.81, 1.01)	0.76 (0.71, 0.82)∗
Constipation	0.75 (0.67, 0.84)∗	0.79 (0.68, 0.91)∗	0.77 (0.67, 0.89)∗	0.61 (0.55, 0.67)∗
GI Toxicity (Composite)	1.08 (1.00, 1.16)∗	1.14 (1.05, 1.23)∗	1.07 (0.99, 1.16)	0.87 (0.83, 0.92)∗
**CARDIAC ADVERSE EVENTS**				
QT Prolongation	0.35 (0.19, 0.66)∗	0.67 (0.30, 1.48)	0.56 (0.26, 1.20)	0.28 (0.15, 0.51)∗
Heart Failure	0.91 (0.77, 1.08)	0.94 (0.75, 1.17)	0.83 (0.67, 1.03)	0.70 (0.60, 0.82)∗
Cardiac Toxicity (Composite)	0.91 (0.82, 1.00)∗	0.95 (0.84, 1.09)	0.87 (0.77, 0.99)∗	0.72 (0.66, 0.79)∗
**OTHER ADVERSE EVENTS**				
Fatigue	0.86 (0.77, 0.96)∗	0.89 (0.77, 1.03)	0.85 (0.74, 0.97)∗	0.71 (0.65, 0.78)∗
Stomatitis	0.49 (0.38, 0.63)∗	0.32 (0.21, 0.50)∗	0.35 (0.24, 0.52)∗	0.36 (0.28, 0.46)∗
Liver Toxicity	1.00 (0.83, 1.20)	0.75 (0.54, 1.03)	0.88 (0.66, 1.18)	0.70 (0.59, 0.84)∗

**Abbreviations:** CI, Confidence Interval; GI, Gastrointestinal; ICU, Intensive Care Unit.

Notes:

•∗ Statistically significant at p < 0.05 (95 % CI does not include 1.0)

•Hazard Ratio represents the overall risk over the study period comparing Abemaciclib versus Palbociclib

•Risk Ratio < 1 indicates lower risk with Abemaciclib; Risk Ratio > 1 indicates higher risk with Abemaciclib

•Risk Ratios shown at 1 year, 3 years, and overall (cumulative risk at end of follow-up)

•Composite outcomes combine related individual outcomes within their respective categories

•Complete time-specific data (1, 3, 6 months, and 5 years) are available in Supplementary Table S2

The survival advantage persisted in the unmatched cohort (HR 0.79, 95 % CI 0.72–0.86), indicating the benefit was not solely due to baseline imbalances. Sensitivity analyses confirmed robustness: on-treatment analysis (HR 0.79, 95 % CI 0.63–0.98), landmark analysis (HR 0.78, 95 % CI 0.69–0.87), and calendar time-restricted analysis (HR 0.89, 95 % CI 0.80–0.99) all showed consistent results (eFig. 2).

### 3.3 Secondary clinical outcomes (hospitalization and survival-related events, Fig. 3, Table 2, and eTable 8)

All-cause hospitalizations were slightly less frequent in the abemaciclib group (cumulative probability: 13.8 % vs 20.2 %, with risk ratio: 0.68, 95 % CI: 0.61–0.77), though the difference did not reach statistical significance based on hazard ratio analysis (HR 0.93, 95 % CI 0.81–1.06; P = 0.27). This apparent discrepancy highlights the complementary nature of these metrics: the non-significant HR suggests similar instantaneous risks of hospitalization over time, while the significantly lower overall risk ratio indicates that cumulatively, fewer patients in the abemaciclib group required hospitalization by the end of follow-up.

ICU admissions also did not differ significantly between groups (10.3 % vs 14.9 %, RR: 0.69, 95 % CI: 0.60–0.79; HR 1.00, 95 % CI 0.85–1.16). Severe infections were significantly less common with abemaciclib compared to palbociclib (15.7 % vs 25.1 %; RR 0.63, 95 % CI 0.56–0.70; HR 0.85, 95 % CI 0.75–0.96). This finding aligns with the lower rates of neutropenia observed with abemaciclib, as neutropenia is a major risk factor for serious infection.

### 3.4 Treatment safety and adverse events (Fig. 3, eTable 8)

The safety profiles of abemaciclib and palbociclib reflected their known distinctions from clinical trials.

● **Hematologic toxicities** were substantially lower with abemaciclib: neutropenia (15.6 % vs 32.2 %), leukopenia (5.6 % vs 10.7 %), and overall hematologic toxicity (39.2 % vs 54.2 %) (all p < 0.001).
● **Gastrointestinal effects** showed mixed patterns. Diarrhea was more common with abemaciclib (24.6 % vs 19.7 %, p < 0.001), while nausea/vomiting (29.1 % vs 38.2 %), constipation (19.6 % vs 32.3 %), and stomatitis (2.9 % vs 8.2 %) were less frequent (all p < 0.05).
● **Other adverse events** generally favored abemaciclib, including lower rates of fatigue (22.0 % vs 30.9 %), headache (12.5 % vs 17.7 %), and pyrexia (8.5 % vs 13.4 %) (all p < 0.05). Alopecia rates were similar between groups (3.3 % vs 4.3 %).
● **Cardiac events** were less frequent with abemaciclib across most categories, including QT prolongation (0.5 % vs 1.7 %, p < 0.001) and overall cardiac toxicity (46.0 % vs 55.6 %, p < 0.05).
  Despite higher diarrhea rates, abemaciclib did not increase hospitalization rates, suggesting effective outpatient management.

### 3.5 Subgroup Analyses (Fig. 4 and eTable 9)

The survival advantage of abemaciclib was generally consistent across patient subgroups (Fig. 4), with some variations in magnitude. No subgroup was identified where palbociclib demonstrated superior outcomes to abemaciclib. The benefit of abemaciclib was particularly pronounced in younger patients (<65 years: HR 0.44, 95 % CI 0.37–0.52), those without brain metastases (HR 0.53, 95 % CI 0.47–0.59), and patients receiving tamoxifen as endocrine partner (HR 0.36, 95 % CI 0.27–0.49). The treatment effect remained consistent across both disease burden subgroups: patients with ≥2 metastatic sites (HR 0.83, 95 % CI 0.70–0.99) and those with visceral metastases (HR 0.81, 95 % CI 0.69–0.96).

**Fig. 3:**
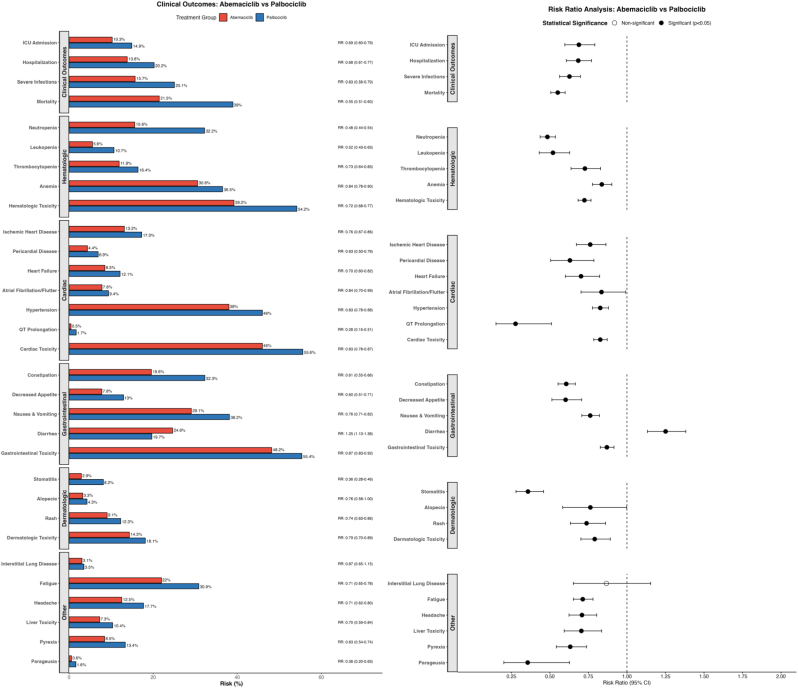
Comparative Adverse Event Profiles of Abemaciclib vs. Palbociclib Dual-panel safety comparison displaying cumulative incidence rates (left panel) and risk ratios with 95 % confidence intervals (right panel). The bar chart provides direct visual comparison of adverse event frequencies between abemaciclib (red) and palbociclib (blue), while the forest plot presents relative risks where values less than 1.0 favor abemaciclib and values greater than 1.0 favor palbociclib. Adverse events are organized by organ system (clinical outcomes, hematologic, cardiac, gastrointestinal, dermatologic, and other) for systematic clinical interpretation. Statistically significant differences (p < 0.05) are marked. (For interpretation of the references to color in this figure legend, the reader is referred to the Web version of this article.)

**Fig. 4:**
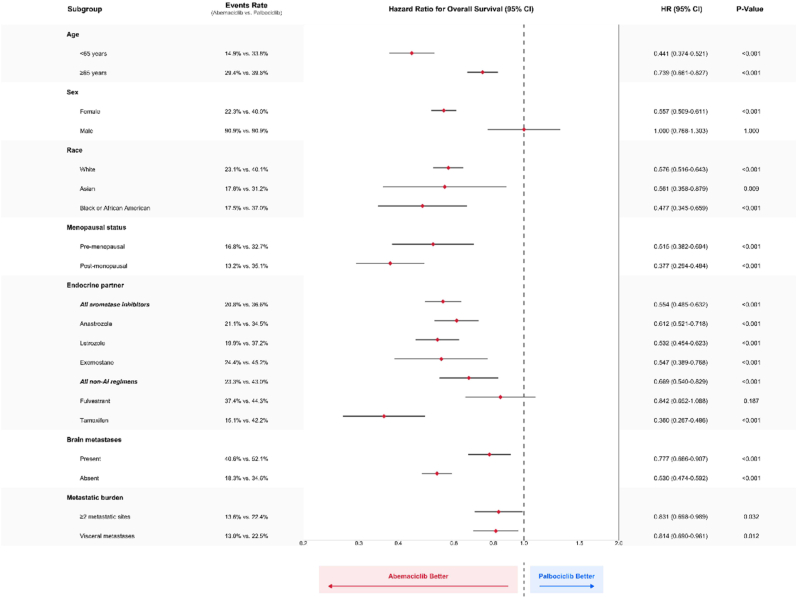
Subgroup Analysis of Overall Survival Forest plot displaying hazard ratios for overall survival across pre-specified patient subgroups. Each horizontal line represents a subgroup comparison, with point estimates showing hazard ratios and line lengths indicating 95 % confidence intervals. The vertical reference line at 1.0 represents no treatment difference; values to the left favor abemaciclib. Subgroups include age categories (<65 vs ≥ 65 years), sex, race (White, Asian, Black/African American), menopausal status, brain metastasis presence, endocrine therapy partners (all aromatase inhibitors, individual AIs, all non-AI regimens, fulvestrant, tamoxifen), and disease burden (≥2 metastatic sites, visceral metastases). Patient numbers, hazard ratios with 95 % confidence intervals, and p-values are provided for each comparison.

E-value analysis confirmed the robustness of our findings in key subgroups. The survival benefit was particularly robust in patients younger than 65 years (E-value 3.96), those receiving tamoxifen (E-value 5.00), and patients without brain metastases (E-value 3.18). These high values suggest the findings in these subgroups are unlikely to be nullified by unmeasured confounding.

### 3.6 Sensitivity analyses and negative control results

Sensitivity analyses supported the robustness of our primary findings. The on-treatment analysis (confirming drug continuity with at least six consecutive doses) yielded a similar hazard ratio for OS (HR 0.79, 95 % CI 0.63–0.98), as did landmark analysis which excluded outcomes within the three months post-treatment initiation (HR 0.78, 95 % CI 0.69–0.87). Regarding calendar time-restricted analysis, reporting similar trend in OS with HR 0.89 (95 % CI: 0.80–0.99) (eTables 3, 4, 5 and eFig. 2).

Negative control analyses showed no significant differences between groups in unrelated events, including kidney stones (HR 0.85, 95 % CI 0.67–1.08), gallbladder stones (HR 1.02, 95 % CI 0.82–1.27), and burns (HR 0.57, 95 % CI 0.26–1.24). These results confirm the effectiveness of our study design in controlling potential confounding (eTable 6).

## 4 Discussion

### 4.1 Main findings and clinical significance

In this large real-world retrospective study emulating a target trial, we found that first-line abemaciclib was associated with significantly improved overall survival compared to palbociclib among patients with HR+/HER2-metastatic breast cancer. After rigorous propensity score matching, abemaciclib demonstrated a 20 % reduction in death risk (HR 0.80, 95 % CI 0.72–0.90), translating to a clinically meaningful 1.0-year prolongation in median survival (6.0 vs 5.0 years). To our knowledge, this represents one of the first large-scale real-world analyses demonstrating a mortality difference between these CDK4/6 inhibitors, suggesting that drug selection in the first-line setting may substantially impact long-term outcomes.

### 4.2 Safety profile insights

The observed safety profiles aligned with known clinical trial data and provide mechanistic insights into the survival advantage. Abemaciclib's significantly lower incidence of neutropenia (15.6 % vs 32.2 %) and severe infections (15.7 % vs 25.1 %) likely enabled more consistent dosing without treatment interruptions. This maintained dose intensity may partially explain the superior overall survival, as optimal CDK4/6 inhibition requires sustained target engagement. While abemaciclib was associated with higher diarrhea rates (24.6 % vs 19.7 %), these events were manageable without increased hospitalizations, indicating effective real-world management strategies [18].

Notably, abemaciclib patients experienced less fatigue (22.0 % vs 30.9 %) and fewer overall hematologic toxicities, potentially reflecting both the drug's distinct mechanism and improved disease control. These quality-of-life advantages, combined with survival benefits, strengthen the clinical case for abemaciclib as first-line therapy.

### 4.3 Comparison with existing literature

Our findings contrast with the recent Flatiron Health analysis by Rugo et al. (2025), which found no significant OS differences between abemaciclib and palbociclib (adjusted HR 0.95, 95 % CI 0.84–1.08) [11]. Several factors may explain this discrepancy. First, our study included a larger abemaciclib cohort (2808 vs 1036 patients) with longer follow-up, providing greater statistical power to detect differences. Second, methodological differences, propensity score matching versus inverse probability weighting, may yield different patient populations for analysis. Third, our inclusion of fulvestrant combinations, in addition to aromatase inhibitors, represents broader real-world practice patterns.

Importantly, both studies observed numerically longer median survival with abemaciclib (our study: 73.5 vs 60.7 months; Rugo et al.: 64.5 vs 54.6 months), suggesting a consistent trend that achieved statistical significance in our larger, longer-duration analysis.

### 4.4 Addressing calendar time bias

A critical methodological consideration in our study was the potential for calendar time bias [19], given palbociclib's earlier FDA approval (2015) versus abemaciclib (2017). Patients treated in the earlier palbociclib era may have had access to fewer effective subsequent-line therapies, potentially confounding survival comparisons [20]. To address this concern, we conducted a calendar time-restricted sensitivity analysis limiting both cohorts to patients diagnosed from 2019 onward, when both CDK4/6 inhibitors and subsequent treatment options were equally established. This analysis demonstrated a consistent survival advantage for abemaciclib (HR 0.89, 95 % CI 0.80–0.99), confirming that our primary findings are not attributable to era-related differences in available therapies.

### 4.5 Study strengths and limitations

Our study's strengths include its large sample size, rigorous target trial emulation methodology, comprehensive propensity score matching achieving excellent covariate balance (all SMD <0.1), and multiple sensitivity analyses [21]. The use of negative control outcomes (kidney stones, burns) that showed no between-group differences validates our study design's ability to control confounding [22].

However, several limitations merit consideration. Despite extensive matching, residual confounding from unmeasured factors (physician preferences, subtle patient frailty differences, tumor genomics) cannot be excluded. The median follow-up in the abemaciclib group (33.7 months) was shorter than the median OS (73.5 months), reflecting its later market approval (2018 vs. 2015 for palbociclib). This limitation requires caution when interpreting long-term survival estimates beyond the median follow-up. However, sensitivity analyses including RMST (which avoids tail extrapolation), calendar time-restricted analysis, and landmark analysis all yielded consistent results, supporting our primary findings.

Real-world data may underestimate some adverse events. Importantly, we lacked information on adverse event severity grading (grade 3/4/5 events), which limits interpretation of the clinical significance of observed toxicity differences, as only high-grade adverse events are clinically meaningful in most cases.

Due to TriNetX database platform constraints, composite disease burden measures (total number of metastatic sites, visceral metastasis status) could not be directly incorporated into propensity score matching, although individual metastatic sites were balanced. These composite measures were evaluated through pre-specified subgroup analyses. Additionally, alternative propensity score methods such as inverse probability weighting could not be implemented due to platform technical limitations. Similarly, the platform does not permit custom multivariable regression modeling on the propensity-matched cohorts, precluding the use of this method as an additional sensitivity analysis.

This analysis focuses solely on first-line therapy and does not capture subsequent treatment sequences that may influence long-term outcomes. Furthermore, the impact of calendar time bias was addressed through sensitivity analyses, but differences in the availability of subsequent therapies between treatment eras may still influence survival outcomes. Furthermore, our exclusion of patients who received CDK4/6 inhibitors in the adjuvant setting may have inadvertently selected for a population with a lower intrinsic risk of recurrence, which should be considered when generalizing our findings.

To assess the potential impact of unmeasured confounding, we calculated an E-value for our primary survival finding. The resulting E-value of 1.81 suggests that a potential unmeasured confounder would need a stronger association with both treatment selection and outcome than most measured prognostic factors in our study to nullify the result. While this does not eliminate the risk of residual bias, it provides a quantitative measure of robustness for our primary finding.

### 4.6 Clinical implications and future directions

These findings have important implications for clinical practice. For patients where maximizing efficacy is paramount—particularly younger patients (<65 years) and those without brain metastases who showed greater benefit in our subgroup analyses—abemaciclib may be the preferred first-line choice. The survival advantage must be weighed against abemaciclib's gastrointestinal toxicity profile, emphasizing the importance of proactive diarrhea management and shared decision-making.

Notably, abemaciclib is the only CDK4/6 inhibitor with FDA approval as monotherapy in heavily pretreated breast cancer and as adjuvant therapy in high-risk early breast cancer [23]. Palbociclib has not shown an OS benefit in its first-line trial (PALOMA-2) [24], whereas abemaciclib has demonstrated OS benefit in the fulvestrant setting (MONARCH 2) [25]. Our findings provide real-world corroboration that abemaciclib may indeed be a more potent agent in the metastatic setting.

Future research should focus on identifying biomarkers that predict differential benefit from specific CDK4/6 inhibitors. Given abemaciclib's unique properties—continuous dosing, brain penetration, and CDK9 inhibition—certain tumor genomic features may confer greater sensitivity to this agent. Additionally, comparative studies including ribociclib would provide a complete picture of CDK4/6 inhibitor relative efficacy.

## 5 Conclusions

In this propensity-matched real-world retrospective analysis, first-line abemaciclib was associated with improved overall survival compared to palbociclib for HR+/HER2-metastatic breast cancer, alongside a distinct but manageable safety profile. While acknowledging the inherent limitations of observational data, these findings contribute to the evidence base for treatment selection in the absence of head-to-head randomized trials. The potential survival benefit of abemaciclib, balanced against its unique toxicity profile, warrants consideration in clinical decision-making, particularly for patients where maximizing long-term survival is the primary treatment goal.

## CRediT authorship contribution statement

**Cho-Hao Lee:** Writing – original draft, Methodology, Investigation, Formal analysis, Data curation, Conceptualization. **Po-Huang Chen:** Writing – original draft, Methodology, Investigation, Formal analysis, Data curation, Conceptualization. **Hong-Jie Jhou:** Investigation, Data curation. **Wei-Cheng Chang:** Resources, Investigation. **Hsin-Yu Chen:** Resources, Investigation. **Li-Ting Kao:** Methodology, Formal analysis. **Tina Yi-Jin Hsieh:** Validation, Investigation. **Ming-Shen Dai:** Writing – review & editing, Supervision, Project administration, Methodology, Funding acquisition, Conceptualization.

## Disclosure

The authors have declared no conflicts of interest.

## IRB statement

This study was deemed exempt from review by the Tri-Service General Hospital Institutional Review Board (approval number: E202516013) due to the use of de-identified data.

## Funding

This study was supported by the 10.13039/501100010425Tri-Service General Hospital (TSGH-D-114168)(PH Chen), 10.13039/501100010425Tri-Service General Hospital (TSGH-D-114074)(CH Lee).

## Declaration of competing interest

The authors declare that they have no known competing financial interests or personal relationships that could have appeared to influence the work reported in this paper.

## Data Availability

The data that support the findings of this study are available from the corresponding author upon reasonable request. Access to the TriNetX Research Network was available through institutional license agreements. De-identified data were used in compliance with relevant data protection and privacy regulations.
