# Supplementary material for: Real-world comparative effectiveness of first-line abemaciclib versus palbociclib in HR+/HER2- metastatic breast cancer: A propensity-matched retrospective analysis
Source: Breast. 2025 Oct 12;84:104597. doi: 10.1016/j.breast.2025.104597 (PMC12550314; doi:10.1016/j.breast.2025.104597)
Supplement: Multimedia component 1 [file mmc1.docx]

# **Supplementary Materials**

**Real-World Comparative Effectiveness of First-Line Abemaciclib versus Palbociclib in HR+/HER2- Metastatic Breast Cancer: A Propensity-Matched Retrospective Analysis**

## Table of Contents

- eTable 1. Target Trial Emulation Framework Comparing Abemaciclib vs Palbociclib
- eTable 2. Diagnostic and Procedure Codes for Study Variables
- eTable 3. Baseline Characteristics Before and After Propensity Score Matching (On-Treatment)
- eTable 4. Results of On-Treatment Cohort Analysis
- eTable 5. Results of Landmark Analysis Cohort Analysis
- eTable 6. Negative Control Outcomes Analysis
- eTable 7. Univariable Cox Proportional Hazards Regression Analysis for Overall Survival (Before Propensity Score Matching)
- eTable 8. Hazard Ratios and Risk Ratios for Clinical Outcomes and Adverse Events Comparing Abemaciclib versus Palbociclib
- eTable 9. Detailed Subgroup Analyses of Abemaciclib versus Palbociclib
- eTable 10. Treatment Patterns and CDK4/6 Inhibitor Usage
- eTable 11. Follow-up Duration and Overall Survival
- eFigure 1. Kaplan−Meier Curves with Restricted Mean Survival Time (RMST) for overall survival (OS)
- eFigure 2. Overall survival plots of sensitivity tests (On-Treatment, and landmark analysis**)**
- eFigure 3. Propensity score density function - Before and after matching
- eMethods. Additional Methodological Details

## eTable 1. Target Trial Emulation Framework Comparing Abemaciclib vs Palbociclib

| **Component** | **Target Trial Protocol** | **Emulated Trial Using Real-World Data** | **Adaptations for Real-World Setting** |
| --- | --- | --- | --- |
| Aim | To compare the effectiveness and safety of abemaciclib versus palbociclib as first-line therapy in HR+/HER2- metastatic breast cancer (mBC) | Same | Real-world data used to emulate the randomized trial framework |
| Eligibility | Adults with HR+/HER2- mBC, no prior CDK4/6i exposure | Same | Patients identified from TriNetX RWD; excluded prior CDK4/6i users to define new-user cohort |
| Treatment Strategies | Random assignment to abemaciclib or palbociclib with endocrine therapy | Physician-assigned treatment | Propensity score matching (1:1) to adjust for baseline confounding |
| Treatment Assignment | Randomization | Propensity score matching | Adjusted for demographics, comorbidities, prior meds, ECOG, BMI |
| Follow-up | From treatment initiation to death, loss to follow-up, or end of study | Same | Median follow-up determined from database censor date (March 2025) |
| Outcome | Primary: Overall survival (OS); Secondary: hospitalization, ICU admission, infections, hematologic AEs | Same | Outcomes defined using ICD-10 and CPT codes (see eTable 2) |
| Statistical Analysis | Kaplan-Meier, Cox regression, and risk ratios | Same | Analyses stratified by matched cohorts with log-rank tests |

## eTable 2. Diagnostic and Procedure Codes for Study Variables

| **Category** | **Variable** | **Coding System** | **Codes** | **Definition** |
| --- | --- | --- | --- | --- |
| **Primary Cancer Diagnosis** | HR+/HER2- mBC | ICD-10 | C50.*, Z17.1 | Hormone receptor-positive, HER2-negative metastatic breast cancer |
| **Medication Exposure** | Abemaciclib | RXNORM/ATC | 1946825/L01EF03 | CDK4/6 inhibitor; Cohort 1 medication |
|  | Palbociclib | RXNORM/ATC | 1601374/L01EF01 | CDK4/6 inhibitor; Cohort 2 medication |
|  | Anastrozole | ATC | L02BG03 | Aromatase inhibitor |
|  | Letrozole | ATC | L02BG04 | Aromatase inhibitor |
|  | Exemestane | ATC | L02BG06 | Aromatase inhibitor |
|  | Fulvestrant | ATC | L02BA03 | Selective estrogen receptor degrader |
|  | Tamoxifen | ATC | L02BA01 | Selective estrogen receptor modulator |
| **Comorbidities** | Hypertension | ICD-10 | I10-I15 | Primary and secondary hypertension |
|  | Diabetes mellitus | ICD-10 | E10-E14 | All types of diabetes |
|  | Hyperlipidemia | ICD-10 | E78.* | Disorders of lipoprotein metabolism |
|  | Heart failure | ICD-10 | I50.* | Systolic, diastolic, or unspecified heart failure |
|  | Coronary artery disease | ICD-10 | I20-I25 | Angina, MI, chronic ischemic heart disease |
|  | Atrial fibrillation/flutter | ICD-10 | I48.* | Arrhythmia conditions |
|  | Stroke/CVA | ICD-10 | I60-I69 | Ischemic and hemorrhagic stroke syndromes |
|  | CKD | ICD-10 | N18.* | All stages of chronic kidney disease |
|  | Liver disease | ICD-10 | K70-K77 | Alcoholic and non-alcoholic liver disease |
|  | Obesity | ICD-10 | E66.* | Based on clinical diagnosis or BMI >30 |
|  | Brain metastases | ICD-10 | C79.31 | Secondary malignant neoplasm of brain |
|  | Other metastatic disease | ICD-10 | C79.* | Secondary neoplasm of other specified sites |
| **Primary Outcome** | Overall Survival | Demographics | Deceased | All-cause mortality, verified against death registry |
| **Secondary Outcomes: Clinical Events** | Hospitalization | CPT/SNOMED | 1013675, 308335008 | Inpatient admission for any cause |
|  | ICU admission | CPT/SNOMED | 99291, 99292, 305351004 | Critical care services or intensive care unit stay |
|  | Severe infection | ICD-10 | A40, A41, J18, R65.20 | Sepsis and pneumonia requiring hospitalization |
| **Secondary Outcomes: Hematologic AEs** | Neutropenia | ICD-10 | D70 | Agranulocytosis and neutropenia |
|  | Leukopenia | ICD-10 | D72.819 | Leukopenia unspecified |
|  | Anemia | ICD-10 | D64 | Other anemias |
|  | Thrombocytopenia | ICD-10 | D69.6 | Unspecified thrombocytopenia |
|  | Hematologic toxicity | ICD-10 | D70, D72.8, D64, D69.* | Composite of all hematologic AEs |
| **Secondary Outcomes: Non-Hematologic AEs** | Diarrhea | ICD-10 | R19.7, K59.1 | Unspecified or functional diarrhea |
|  | Nausea & Vomiting | ICD-10 | R11 | Nausea and vomiting |
|  | Stomatitis | ICD-10 | K12 | Stomatitis and related lesions |
|  | Constipation | ICD-10 | K59.0 | Constipation |
|  | Decreased Appetite | ICD-10 | R63.0 | Anorexia |
|  | Dry Skin | ICD-10 | L85.3 | Xerosis cutis |
|  | Rash | ICD-10 | R21 | Nonspecific skin eruption |
|  | Dermatologic Toxicity | ICD-10 | L29.9, L65, L85.3, R21 | Grouped skin AEs |
|  | Fatigue | ICD-10 | R53.83 | Other fatigue |
|  | Headache | ICD-10 | R51 | Headache |
|  | Parageusia | ICD-10 | R43.2 | Taste distortion |
|  | Pyrexia | ICD-10 | R50.9 | Fever |
|  | Cardiac Toxicity | ICD-10 | I48.91, I50, I20-I25, I31, I45.81 | Grouped cardiac events |
|  | QT Prolongation | ICD-10 | I45.81 | Long QT syndrome |
|  | Heart Failure | ICD-10 | I50 | Heart failure |
|  | Atrial Fibrillation/Flutter | ICD-10 | I48 | AF and flutter |
|  | Ischemic Heart Disease | ICD-10 | I20-I25 | Angina, MI, chronic IHD |
|  | Pericardial Disease | ICD-10 | I31 | Pericarditis and pericardial effusion |
|  | Interstitial Lung Disease | ICD-10 | J84.10 | Pulmonary fibrosis |
|  | Liver Toxicity | ICD-10 | R74.0 | Elevated liver enzymes |
| **Negative Control Outcomes** | Kidney Stones | ICD-10 | N20.0, N20.1, N20.2 | Renal/ureteral calculi |
|  | Gallbladder Stones | ICD-10 | K80 | Cholelithiasis |
|  | Burn | ICD-10 | T20-T31 | Thermal/chemical burn |

**Abbreviations:** AEs, adverse events; ATC, Anatomical Therapeutic Chemical; BMI, body mass index; CPT, Current Procedural Terminology; CKD, chronic kidney disease; ICD-10, International Classification of Diseases, Tenth Revision; ICU, intensive care unit; MI, myocardial infarction; RXNORM, standardized nomenclature for clinical drugs; SNOMED, Systematized Nomenclature of Medicine.

## eTable 3. Baseline Characteristics Before and After Propensity Score Matching of On-Treatment Design Cohort

| Characteristics | Before Propensity Score Matching | | | | After Propensity Score Matching | | | |
| --- | --- | --- | --- | --- | --- | --- | --- | --- |
|  | Abemaciclib (n=719) | Palbociclib (n=2,688) | P Value | SMD | Abemaciclib (n=693) | Palbociclib  (n=693) | P Value | SMD |
| **Demographics** |  |  |  |  |  |  |  |  |
| Age at index, mean (SD), y | 60.3 (12.9) | 63.5 (12.4) | <.001 | 0.248 | 60.7 (12.9) | 61.2 (12.6) | 0.475 | 0.038 |
| **Sex, No. (%)** |  |  |  |  |  |  |  |  |
| Female | 711 (98.9) | 2,652 (98.7) | 0.633 | 0.021 | 685 (98.8) | 683 (98.6) | 0.635 | 0.025 |
| Male | 10 (1.1) | 36 (1.3) | 0.915 | 0.004 | 10 (1.1) | 10 (1.4) | >.999 | <0.001 |
| Unknown | 0 (0.0) | 0 (0.0) | -- | -- | 0 (0.0) | 0 (0.0) | -- | -- |
| **Race/Ethnicity, No. (%)** |  |  |  |  |  |  |  |  |
| White | 478 (66.5) | 1,969 (73.3) | <.001 | 0.148 | 468 (67.5) | 477 (68.8) | 0.604 | 0.028 |
| Black or African American | 87 (12.1) | 358 (13.3) | 0.389 | 0.037 | 86 (12.4) | 76 (11.0) | 0.403 | 0.045 |
| Asian | 67 (9.3) | 103 (3.8) | <.001 | 0.223 | 53 (7.6) | 52 (7.5) | 0.919 | 0.005 |
| Unknown | 44 (6.1) | 119 (4.4) | 0.059 | 0.076 | 44 (6.3) | 42 (6.1) | 0.824 | 0.012 |
| **Comorbidities, No. (%)** |  |  |  |  |  |  |  |  |
| Diabetes mellitus | 113 (15.7) | 376 (14.0) | 0.24 | 0.049 | 108 (15.6) | 110 (15.9) | 0.883 | 0.008 |
| Hypertensive diseases | 248 (34.5) | 985 (36.6) | 0.286 | 0.045 | 240 (34.6) | 238 (34.3) | 0.91 | 0.006 |
| Ischemic heart diseases | 52 (7.2) | 183 (6.8) | 0.69 | 0.017 | 47 (6.8) | 57 (8.2) | 0.308 | 0.055 |
| Heart failure | 37 (5.1) | 133 (4.9) | 0.828 | 0.009 | 36 (5.2) | 39 (5.6) | 0.722 | 0.019 |
| Chronic obstructive pulmonary disease | 19 (2.6) | 114 (4.2) | 0.049 | 0.088 | 19 (2.7) | 17 (2.5) | 0.736 | 0.018 |
| Chronic kidney disease | 70 (9.7) | 251 (9.3) | 0.746 | 0.014 | 66 (9.5) | 64 (9.2) | 0.854 | 0.01 |
| Cerebral infarction | 14 (1.9) | 59 (2.2) | 0.684 | 0.017 | 14 (2.0) | 14 (2.0) | >.999 | <0.001 |
| Cirrhosis of liver | 10 (1.4) | 15 (0.6) | 0.02 | 0.085 | 10 (1.4) | 10 (1.4) | >.999 | <0.001 |
| Systemic lupus erythematosus | 10 (1.4) | 10 (0.4) | 0.001 | 0.109 | 10 (1.4) | 10 (1.4) | >.999 | <0.001 |
| Psoriasis | 10 (1.4) | 27 (1.0) | 0.375 | 0.036 | 10 (1.4) | 10 (1.4) | >.999 | <0.001 |
| Overweight/obesity | 147 (20.4) | 375 (14.0) | <.001 | 0.173 | 134 (19.3) | 137 (19.8) | 0.839 | 0.011 |
| **Metastatic Sites, No. (%)** |  |  |  |  |  |  |  |  |
| Bone metastases | 58 (8.1) | 136 (5.1) | 0.002 | 0.122 | 53 (7.6) | 51 (7.4) | 0.838 | 0.011 |
| Brain metastases | 141 (19.6) | 330 (12.3) | <.001 | 0.198 | 128 (18.5) | 124 (17.9) | 0.779 | 0.015 |
| Lung metastases | 125 (17.4) | 293 (10.9) | <.001 | 0.186 | 114 (16.5) | 110 (15.9) | 0.767 | 0.016 |
| Liver metastases | 23 (3.2) | 54 (2.0) | 0.061 | 0.073 | 21 (3.0) | 20 (2.9) | 0.885 | 0.008 |
| Peritoneal metastases | 537 (74.7) | 1,260 (46.9) | <.001 | 0.59 | 491 (70.9) | 472 (68.1) | 0.276 | 0.059 |
| **Concomitant Endocrine Therapy, No. (%)** |  |  |  |  |  |  |  |  |
| Tamoxifen | 95 (13.2) | 330 (12.3) | 0.5 | 0.028 | 91 (13.1) | 92 (13.3) | 0.937 | 0.004 |
| Fulvestrant | 123 (17.1) | 563 (20.9) | 0.023 | 0.098 | 118 (17.0) | 118 (17.0) | >.999 | <0.001 |
| Exemestane | 76 (10.6) | 310 (11.5) | 0.47 | 0.031 | 74 (10.7) | 62 (8.9) | 0.279 | 0.058 |
| Letrozole | 268 (37.3) | 1,507 (56.1) | <.001 | 0.383 | 265 (38.2) | 265 (38.2) | >.999 | <0.001 |
| Anastrozole | 277 (38.5) | 665 (24.7) | <.001 | 0.3 | 261 (37.7) | 246 (35.5) | 0.403 | 0.045 |
| **Clinical Measurements** |  |  |  |  |  |  |  |  |
| ECOG performance status, mean (SD) | 0.3 (0.6) | 0.8 (1.3) | 0.152 | 0.526 | 0.2 (0.6) | 1.1 (1.4) | 0.045 | 0.876 |
| Body mass index, mean (SD), kg/m² | 29.2 (7.2) | 28.8 (7.2) | 0.322 | 0.047 | 29.2 (7.2) | 29.4 (7.4) | 0.703 | 0.022 |
| **Social Determinants of Health, No. (%)** |  |  |  |  |  |  |  |  |
| Problems related to social environment | 10 (1.4) | 14 (0.5) | 0.013 | 0.09 | 10 (1.4) | 10 (1.4) | >.999 | <0.001 |
| Problems related to housing/economic circumstances | 11 (1.5) | 28 (1.0) | 0.274 | 0.043 | 10 (1.4) | 10 (1.4) | >.999 | <0.001 |
| Problems related to employment | 10 (1.4) | 0 (0.0) | <.001 | 0.168 | 0 (0.0) | 0 (0.0) | -- | -- |
| Problems related to education/literacy | 10 (1.4) | 10 (0.4) | 0.001 | 0.109 | 10 (1.4) | 10 (1.4) | >.999 | <0.001 |

**Abbreviations:** SMD, standardized mean difference; SD, standard deviation; BMI, body mass index; ECOG, Eastern Cooperative Oncology Group.

**Note:** After propensity score matching, all standardized mean differences were <0.1, indicating successful balancing of baseline characteristics between the two treatment groups.

## eTable 4. Results of On-Treatment Design Cohort Analysis

| **Outcome** | **Patients (Abe/Pal)** | **Events (Abe/Pal)** | **Risk Ratio (95% CI)** | **Hazard Ratio (95% CI)** | **P-value (Log-Rank)** |
| --- | --- | --- | --- | --- | --- |
| Overall Survival | 693/693 | 120/257 | 0.47 (0.39, 0.56) | 0.79 (0.63, 0.98) | 0.034* |
| Hospitalization | 693/693 | 46/60 | NA | 1.08 (0.73, 1.59) | 0.713 |
| ICU admission | 693/693 | 61/103 | 0.59 (0.44, 0.80) | 0.91 (0.66, 1.26) | 0.559 |
| Severe infections | 693/693 | 109/178 | 0.61 (0.49, 0.76) | 0.88 (0.69, 1.12) | 0.290 |
| Neutropenia | 693/693 | 133/278 | 0.48 (0.40, 0.57) | 0.50 (0.41, 0.61) | <0.001* |
| Leukopenia | 693/693 | 35/90 | 0.39 (0.27, 0.57) | 0.47 (0.31, 0.69) | <0.001* |
| Anemia | 693/693 | 204/243 | 0.84 (0.72, 0.98) | 1.11 (0.92, 1.34) | 0.284 |
| Thrombocytopenia | 693/693 | 74/114 | 0.65 (0.49, 0.85) | 0.91 (0.68, 1.23) | 0.546 |
| Fatigue | 693/693 | 173/231 | 0.75 (0.63, 0.89) | 0.92 (0.75, 1.12) | 0.401 |
| Nausea & Vomiting | 693/693 | 215/300 | 0.72 (0.62, 0.82) | 0.88 (0.74, 1.05) | 0.166 |
| Diarrhea | 693/693 | 213/147 | 1.45 (1.21, 1.74) | 2.12 (1.71, 2.63) | <0.001* |
| Stomatitis | 693/693 | 27/65 | 0.42 (0.27, 0.64) | 0.60 (0.38, 0.94) | 0.024* |
| Alopecia | 693/693 | 30/37 | 0.81 (0.51, 1.30) | 1.16 (0.71, 1.91) | 0.550 |
| Rash | 693/693 | 76/116 | 0.66 (0.50, 0.86) | 0.86 (0.64, 1.16) | 0.327 |
| Headache | 693/693 | 109/147 | 0.74 (0.59, 0.93) | 0.95 (0.74, 1.22) | 0.685 |
| Constipation | 693/693 | 153/264 | 0.58 (0.49, 0.69) | 0.72 (0.59, 0.88) | 0.001* |
| Liver Toxicity | 693/693 | 56/85 | 0.66 (0.48, 0.91) | 0.93 (0.66, 1.31) | 0.665 |
| Interstitial Lung Disease | 693/693 | 32/28 | 1.14 (0.70, 1.88) | 1.62 (0.96, 2.71) | 0.066 |
| Hypertension | 693/693 | 237/300 | 0.79 (0.69, 0.90) | 0.83 (0.70, 0.99) | 0.034* |
| Atrial Fibrillation/Flutter | 693/693 | 52/60 | 0.87 (0.61, 1.24) | 1.14 (0.78, 1.67) | 0.485 |
| Heart Failure | 693/693 | 59/91 | 0.65 (0.48, 0.88) | 0.88 (0.63, 1.23) | 0.467 |
| Ischemic Heart Disease | 693/693 | 82/118 | 0.70 (0.54, 0.90) | 0.91 (0.68, 1.21) | 0.514 |
| Pericardial Disease | 693/693 | 29/54 | 0.54 (0.35, 0.83) | 0.81 (0.51, 1.28) | 0.361 |
| QT Prolongation | 693/693 | 10/17 | 0.59 (0.27, 1.28) | 0.22 (0.07, 0.76) | 0.009* |
| Decreased Appetite | 693/693 | 50/87 | 0.58 (0.41, 0.80) | 0.78 (0.54, 1.11) | 0.159 |
| Pyrexia | 693/693 | 57/131 | 0.44 (0.33, 0.58) | 0.58 (0.42, 0.79) | 0.001* |
| Hematologic toxicity | 693/693 | 280/408 | 0.69 (0.62, 0.77) | 0.73 (0.63, 0.85) | <0.001* |
| Cardiac Toxicity | 693/693 | 142/211 | 0.67 (0.56, 0.81) | 0.87 (0.70, 1.08) | 0.204 |
| Dermatologic Toxicity | 693/693 | 120/171 | 0.70 (0.57, 0.86) | 0.93 (0.73, 1.18) | 0.533 |
| Gastrointestinal Toxicity | 693/693 | 380/434 | 0.88 (0.80, 0.96) | 1.14 (0.99, 1.31) | 0.072 |
| Kidney stones | 693/693 | 24/54 | 0.44 (0.28, 0.71) | 0.56 (0.34, 0.90) | 0.016* |
| Gallbladder stone | 693/693 | 43/51 | 0.84 (0.57, 1.25) | 1.15 (0.76, 1.73) | 0.515 |
| Burn | 693/693 | 10/10 | 1.00 (0.42, 2.39) | 1.01 (0.28, 3.67) | 0.992 |

**Notes:**

- For each outcome, there were 693 patients in each cohort after propensity score matching
- Risk ratio < 1 indicates lower risk in the Abemaciclib group compared to the Palbociclib group
- Hazard ratio < 1 indicates lower hazard (better outcome) in the Abemaciclib group
- Statistically significant p-values (p<0.05)
- Abe = Abemaciclib; Pal = Palbociclib; CI = Confidence Interval

## eTable 5. Results of Landmark Analysis Cohort Analysis (exclude patients who died/censored within 3 months)

| **Outcome** | **Patients (Abe/Pal)** | **Events (Abe/Pal)** | **Risk Ratio (95% CI)** | **Hazard Ratio (95% CI)** | **P-value (Log-Rank)** |
| --- | --- | --- | --- | --- | --- |
| Overall Survival | 2,198/2,198 | 458/855 | 0.54 (0.49, 0.59) | 0.78 (0.69, 0.87) | <0.001* |
| Hospitalization | 2,198/2,198 | 127/174 | NA | 0.96 (0.76, 1.20) | 0.698 |
| ICU admission | 2,198/2,198 | 226/309 | 0.73 (0.62, 0.86) | 1.03 (0.87, 1.23) | 0.729 |
| Severe infections | 2,198/2,198 | 364/526 | 0.69 (0.61, 0.78) | 0.94 (0.82, 1.07) | 0.352 |
| Neutropenia | 2,198/2,198 | 333/724 | 0.46 (0.41, 0.52) | 0.49 (0.43, 0.55) | <0.001* |
| Leukopenia | 2,198/2,198 | 123/249 | 0.49 (0.40, 0.61) | 0.60 (0.48, 0.74) | <0.001* |
| Anemia | 2,198/2,198 | 635/799 | 0.80 (0.73, 0.87) | 1.01 (0.91, 1.12) | 0.920 |
| Thrombocytopenia | 2,198/2,198 | 252/380 | 0.66 (0.57, 0.77) | 0.85 (0.73, 1.00) | 0.056 |
| Fatigue | 2,198/2,198 | 508/730 | 0.70 (0.63, 0.77) | 0.82 (0.74, 0.92) | 0.001* |
| Nausea & Vomiting | 2,198/2,198 | 659/894 | 0.74 (0.68, 0.80) | 0.90 (0.81, 0.99) | 0.038* |
| Diarrhea | 2,198/2,198 | 547/460 | 1.19 (1.07, 1.33) | 1.69 (1.49, 1.92) | <0.001* |
| Stomatitis | 2,198/2,198 | 65/200 | 0.33 (0.25, 0.43) | 0.42 (0.31, 0.55) | <0.001* |
| Alopecia | 2,198/2,198 | 70/92 | 0.76 (0.56, 1.03) | 1.01 (0.73, 1.38) | 0.964 |
| Rash | 2,198/2,198 | 197/276 | 0.71 (0.60, 0.85) | 0.94 (0.78, 1.14) | 0.536 |
| Headache | 2,198/2,198 | 271/397 | 0.68 (0.59, 0.79) | 0.89 (0.76, 1.04) | 0.131 |
| Constipation | 2,198/2,198 | 459/733 | 0.63 (0.57, 0.69) | 0.77 (0.68, 0.86) | <0.001* |
| Liver Toxicity | 2,198/2,198 | 153/236 | 0.65 (0.53, 0.79) | 0.90 (0.73, 1.10) | 0.306 |
| Interstitial Lung Disease | 2,198/2,198 | 75/70 | 1.07 (0.78, 1.48) | 1.58 (1.13, 2.21) | 0.007* |
| Hypertension | 2,198/2,198 | 858/990 | 0.87 (0.81, 0.93) | 0.97 (0.89, 1.06) | 0.519 |
| Atrial Fibrillation/Flutter | 2,198/2,198 | 176/187 | 0.94 (0.77, 1.15) | 1.19 (0.97, 1.47) | 0.103 |
| Heart Failure | 2,198/2,198 | 224/292 | 0.77 (0.65, 0.90) | 0.98 (0.82, 1.16) | 0.781 |
| Ischemic Heart Disease | 2,198/2,198 | 296/363 | 0.82 (0.71, 0.94) | 1.03 (0.88, 1.20) | 0.703 |
| Pericardial Disease | 2,198/2,198 | 94/167 | 0.56 (0.44, 0.72) | 0.77 (0.60, 1.00) | 0.046* |
| QT Prolongation | 2,198/2,198 | 12/39 | 0.31 (0.16, 0.59) | 0.38 (0.20, 0.73) | 0.003* |
| Parageusia | 2,198/2,198 | 13/30 | 0.43 (0.23, 0.83) | 0.67 (0.35, 1.30) | 0.235 |
| Decreased Appetite | 2,198/2,198 | 173/275 | 0.63 (0.53, 0.75) | 0.81 (0.67, 0.98) | 0.033* |
| Pyrexia | 2,198/2,198 | 177/314 | 0.56 (0.47, 0.67) | 0.72 (0.60, 0.87) | 0.001* |
| Dry skin | 2,198/2,198 | 0/0 | N/A | N/A | 1.000 |
| Hematologic toxicity | 2,198/2,198 | 826/1,215 | 0.68 (0.64, 0.73) | 0.72 (0.66, 0.79) | <0.001* |
| Cardiac Toxicity | 2,198/2,198 | 515/679 | 0.76 (0.69, 0.84) | 0.94 (0.83, 1.05) | 0.270 |
| Dermatologic Toxicity | 2,198/2,198 | 311/415 | 0.75 (0.66, 0.86) | 0.97 (0.84, 1.13) | 0.729 |
| Gastrointestinal Toxicity | 2,198/2,198 | 1,093/1,255 | 0.87 (0.82, 0.92) | 1.08 (1.00, 1.17) | 0.066 |
| Kidney stones | 2,198/2,198 | 91/117 | 0.78 (0.60, 1.02) | 1.00 (0.75, 1.31) | 0.973 |
| Gallbladder stone | 2,198/2,198 | 124/150 | 0.83 (0.66, 1.04) | 1.08 (0.85, 1.37) | 0.553 |
| Burn | 2,198/2,198 | 10/14 | 0.71 (0.32, 1.61) | 0.93 (0.40, 2.19) | 0.875 |

**Notes:**

- For each outcome, there were 2,198 patients in each cohort after propensity score matching
- Risk ratio < 1 indicates lower risk in the Abemaciclib group compared to the Palbociclib group
- Hazard ratio < 1 indicates lower hazard (better outcome) in the Abemaciclib group
- Statistically significant p-values (p<0.05)
- Abe = Abemaciclib; Pal = Palbociclib; CI = Confidence Interval; N/A = Not applicable

## eTable 6. Negative Control Outcomes Analysis

| **Analysis Type** | **Outcome** | **Events (Abemaciclib vs Palbociclib)** | **After PSM, HR (95% CI)** | **P Value (After PSM)** |
| --- | --- | --- | --- | --- |
| **As-Started Analysis** | Kidney Stone | 91/117 | 0.85 (0.67, 1.08) | 0.184 |
|  | Gallbladder Stone | 124/150 | 1.02 (0.82, 1.27) | 0.860 |
|  | Burn | 10/14 | 0.93 (0.40, 2.19) | 0.875 |
| **On-Treatment Analysis** | Kidney Stone | 34/54 | 0.76 (0.34, 1.30) | 0.156 |
|  | Gallbladder Stone | 43/51 | 1.15 (0.76, 1.73) | 0.515 |
|  | Burn | 10/10 | 1.01 (0.28, 3.67) | 0.992 |

**Abbreviations:** CI, confidence interval; HR, hazard ratio; PSM, propensity score matching.

**Note:** Negative control outcomes assess potential unmeasured confounding; HRs near 1.0 suggest minimal residual bias. P values were calculated using log-rank test. * Statistically significant (p<0.05).

## eTable 7. Univariable Cox Proportional Hazards Regression Analysis for Overall Survival (Before Propensity Score Matching)

| **Variable** | **Hazard Ratio** | **95% CI** | **P value** |
| --- | --- | --- | --- |
| **Treatment Group** |  |  |  |
| **Abemaciclib** vs. Palbociclib | 0.63 | 0.43–0.93 | 0.020 |
| **Demographics** |  |  |  |
| Age at index (per year) | 1.01 | 1.01–1.01 | <0.001 |
| Male sex | 1.12 | 0.88–1.43 | 0.358 |
| BMI percentile | 1.08 | 0.95–1.23 | 0.220 |
| **Comorbidities** |  |  |  |
| Diabetes mellitus | 1.09 | 1.01–1.18 | 0.022 |
| Hypertensive diseases | 1.03 | 0.97–1.10 | 0.336 |
| Ischemic heart diseases | 1.09 | 0.99–1.19 | 0.079 |
| Heart failure | 1.28 | 1.15–1.43 | <0.001 |
| COPD | 1.29 | 1.16–1.44 | <0.001 |
| Chronic kidney disease | 1.34 | 1.21–1.48 | <0.001 |
| Cerebral infarction | 1.15 | 0.97–1.36 | 0.119 |
| Liver cirrhosis | 1.66 | 1.35–2.04 | <0.001 |
| **Endocrine Therapy** |  |  |  |
| Aromatase inhibitors | 1.23 | 1.17–1.30 | <0.001 |
| Tamoxifen | 1.17 | 1.07–1.27 | <0.001 |
| Fulvestrant | 1.42 | 1.32–1.52 | <0.001 |
| **Metastatic Sites** |  |  |  |
| Lung | 1.00 | 0.92–1.08 | 0.954 |
| Liver | 1.90 | 1.77–2.05 | <0.001 |
| Brain | 1.55 | 1.39–1.72 | <0.001 |
| Peritoneum | 1.63 | 1.40–1.90 | <0.001 |
| Bone | 1.22 | 1.16–1.29 | <0.001 |
| Other sites | 0.93 | 0.85–1.03 | 0.159 |

**Abbreviations:** HR, hazard ratio; CI, confidence interval; COPD, chronic obstructive pulmonary disease; BMI, body mass index.

**eTable 8. Hazard Ratios, Risk Ratios, and E-values for All Clinical Outcomes: Abemaciclib versus Palbociclib**

## **Primary Outcome and Clinical Events**

| **Outcome** | **HR (95% CI)** | **E-value (HR)** | **RR 1m** | **RR 3m** | **RR 6m** | **RR 1yr** | **RR 3yr** | **RR 5yr** | **RR Overall** |
| --- | --- | --- | --- | --- | --- | --- | --- | --- | --- |
| **Overall Survival** | 0.80 (0.72, 0.90)* | 1.81 | 1.10 | 1.01 | 0.90 | 0.78* | 0.65* | 0.59* | 0.55* |
| **Hospitalization** | 0.93 (0.81, 1.06) | — | 1.27 | 0.94 | 1.13 | 0.93 | 0.73* | 0.71* | 0.68* |
| **ICU Admission** | 1.00 (0.85, 1.16) | — | 1.52 | 1.31 | 1.11 | 1.33* | 0.94 | 0.74* | 0.69* |
| **Severe Infections** | 0.85 (0.75, 0.96)* | 1.63 | 0.72 | 0.82 | 0.90 | 0.82* | 0.72* | 0.64* | 0.63* |

## **Hematologic Adverse Events**

| **Outcome** | **HR (95% CI)** | **E-value (HR)** | **RR 1m** | **RR 3m** | **RR 6m** | **RR 1yr** | **RR 3yr** | **RR 5yr** | **RR Overall** |
| --- | --- | --- | --- | --- | --- | --- | --- | --- | --- |
| **Neutropenia** | 0.52 (0.47, 0.59)* | 3.26 | 0.50* | 0.44* | 0.53* | 0.51* | 0.51* | 0.49* | 0.48* |
| **Leukopenia** | 0.63 (0.52, 0.76)* | 2.55 | 0.67 | 0.52* | 0.57* | 0.57* | 0.60* | 0.62* | 0.52* |
| **Anemia** | 1.08 (0.98, 1.18) | — | 1.27* | 1.10 | 1.07 | 1.07 | 0.95 | 0.86* | 0.84* |
| **Thrombocytopenia** | 0.97 (0.84, 1.12) | — | 1.09 | 1.03 | 1.01 | 1.05 | 0.74* | 0.69* | 0.73* |
| **Hematologic Toxicity (Composite)** | 0.79 (0.73, 0.86)* | 1.85 | 0.87 | 0.74* | 0.80* | 0.78* | 0.76* | 0.74* | 0.72* |

## **Gastrointestinal Adverse Events**

| **Outcome** | **HR (95% CI)** | **E-value (HR)** | **RR 1m** | **RR 3m** | **RR 6m** | **RR 1yr** | **RR 3yr** | **RR 5yr** | **RR Overall** |
| --- | --- | --- | --- | --- | --- | --- | --- | --- | --- |
| **Diarrhea** | 1.83 (1.63, 2.05)* | 3.18 | 3.52* | 2.83* | 2.52* | 2.10* | 1.48* | 1.32* | 1.25* |
| **Nausea & Vomiting** | 0.95 (0.86, 1.04) | — | 0.99 | 0.94 | 1.03 | 0.90 | 0.78* | 0.76* | 0.76* |
| **Constipation** | 0.75 (0.67, 0.84)* | 2.00 | 0.84 | 0.72* | 0.79* | 0.77* | 0.69* | 0.63* | 0.61* |
| **Decreased Appetite** | 0.80 (0.67, 0.95)* | 1.81 | 1.11 | 1.12 | 0.86 | 0.76* | 0.72* | 0.65* | 0.60* |
| **GI Toxicity (Composite)** | 1.08 (1.00, 1.16)* | 1.37 | 1.19* | 1.11* | 1.14* | 1.07 | 0.92* | 0.88* | 0.87* |

## **Dermatologic Toxicity**

| **Outcome** | **HR (95% CI)** | **E-value (HR)** | **RR 1m** | **RR 3m** | **RR 6m** | **RR 1yr** | **RR 3yr** | **RR 5yr** | **RR Overall** |
| --- | --- | --- | --- | --- | --- | --- | --- | --- | --- |
| **Stomatitis** | 0.49 (0.38, 0.63)* | 3.50 | 0.56 | 0.33* | 0.32* | 0.35* | 0.33* | 0.36* | 0.36* |
| **Rash** | 1.01 (0.85, 1.19) | — | 0.62 | 0.74 | 0.80 | 0.77* | 0.81* | 0.75* | 0.74* |
| **Alopecia** | 1.04 (0.79, 1.38) | — | 1.00 | 1.17 | 1.00 | 0.91 | 0.94 | 0.83 | 0.76 |
| **Dermatologic Toxicity (Composite)** | 1.06 (0.92, 1.21) | — | 0.71 | 0.86 | 0.81 | 0.85 | 0.88 | 0.81* | 0.79* |

## **Cardiac Adverse Events**

| **Outcome** | **HR (95% CI)** | **E-value (HR)** | **RR 1m** | **RR 3m** | **RR 6m** | **RR 1yr** | **RR 3yr** | **RR 5yr** | **RR Overall** |
| --- | --- | --- | --- | --- | --- | --- | --- | --- | --- |
| **QT Prolongation** | 0.35 (0.19, 0.66)* | 5.14 | 1.00 | 1.00 | 0.67 | 0.56 | 0.52* | 0.26* | 0.28* |
| **Heart Failure** | 0.91 (0.77, 1.08) | — | 0.71 | 0.82 | 0.94 | 0.83 | 0.79* | 0.71* | 0.70* |
| **Atrial Fibrillation/Flutter** | 1.08 (0.90, 1.30) | — | 1.07 | 1.13 | 1.15 | 1.10 | 0.97 | 0.98 | 0.84* |
| **Ischemic Heart Disease** | 0.99 (0.86, 1.14) | — | 1.05 | 1.02 | 0.90 | 0.96 | 0.89 | 0.81* | 0.76* |
| **Pericardial Disease** | 0.88 (0.70, 1.11) | — | 1.50 | 1.17 | 1.20 | 0.84 | 0.81 | 0.71* | 0.63* |
| **Hypertension** | 0.92 (0.85, 1.00) | — | 0.93 | 0.98 | 0.97 | 0.97 | 0.92* | 0.88* | 0.83* |
| **Cardiac Toxicity (Composite)** | 0.91 (0.82, 1.00)* | 1.43 | 0.99 | 0.94 | 0.95 | 0.87* | 0.84* | 0.77* | 0.72* |

## **Other Outcomes**

| **Outcome** | **HR (95% CI)** | **E-value (HR)** | **RR 1m** | **RR 3m** | **RR 6m** | **RR 1yr** | **RR 3yr** | **RR 5yr** | **RR Overall** |
| --- | --- | --- | --- | --- | --- | --- | --- | --- | --- |
| **Liver Toxicity** | 1.00 (0.83, 1.20) | — | 0.80 | 0.93 | 0.75 | 0.88 | 0.89 | 0.73* | 0.70* |
| **Interstitial Lung Disease** | 1.29 (0.96, 1.74) | — | 1.00 | 1.00 | 1.13 | 1.18 | 1.17 | 1.19 | 0.87 |
| **Fatigue** | 0.86 (0.77, 0.96)* | 1.60 | 0.91 | 0.88 | 0.89 | 0.85* | 0.80* | 0.71* | 0.71* |
| **Headache** | 0.91 (0.79, 1.05) | — | 1.21 | 0.95 | 0.81 | 0.87 | 0.77* | 0.72* | 0.71* |
| **Pyrexia** | 0.86 (0.73, 1.01) | — | 0.90 | 0.85 | 0.74* | 0.82 | 0.73* | 0.63* | 0.63* |

**Abbreviations:** CI, Confidence Interval; HR, Hazard Ratio; RR, Risk Ratio.

**Notes:**

- * Statistically significant at p < 0.05 (95% CI does not include 1.0)
- HR: Hazard ratio from Cox proportional hazards model
- E-value (HR): E-value for hazard ratio point estimate (only shown for significant HRs). Higher values indicate greater robustness to unmeasured confounding
- RR: Risk ratios at 1 month, 3 months, 6 months, 1 year, 3 years, 5 years, and overall (end of follow-up)
- Values < 1.0 favor Abemaciclib; Values > 1.0 favor Palbociclib
- "—" indicates non-significant HR (E-value not calculated)

## **eTable 9. Subgroup Analysis of Overall Survival in Patients Treated with Abemaciclib versus Palbociclib (Propensity Score-Matched Cohort, n=2,768 per group)**

| **Variable** | **Subgroup** | **n per group** | **Abemaciclib Events (n)** | **Palbociclib Events (n)** | **HR (95% CI)** | **P-Value** | **E-value** |
| --- | --- | --- | --- | --- | --- | --- | --- |
| **Age** | <65 years | 1,306 | 195 | 441 | 0.441 (0.374-0.521) | <0.001 | 3.96 |
|  | ≥65 years | 1,462 | 430 | 582 | 0.739 (0.661-0.827) | <0.001 | 2.04 |
| **Menopausal status** | Premenopausal | 327 | 55 | 107 | 0.515 (0.382-0.694) | <0.001 | 3.29 |
|  | Postmenopausal | 562 | 74 | 197 | 0.377 (0.294-0.484) | <0.001 | 4.75 |
| **Sex** | Female | 2,629 | 586 | 1,051 | 0.557 (0.509-0.611) | <0.001 | 2.99 |
|  | Male | 33 | 30 | 30 | 1.000 (0.768-1.303) | 1 | 1 |
| **Race** | White | 1,875 | 433 | 751 | 0.576 (0.516-0.643) | <0.001 | 2.87 |
|  | Asian | 157 | 28 | 49 | 0.561 (0.358-0.879) | 0.009 | 2.96 |
|  | Black or African American | 359 | 63 | 133 | 0.477 (0.345-0.659) | <0.001 | 3.61 |
| **Brain metastases** | Present | 217 | 88 | 113 | 0.777 (0.666-0.907) | <0.001 | 1.89 |
|  | Absent | 2,551 | 468 | 883 | 0.530 (0.474-0.592) | <0.001 | 3.18 |
| **Endocrine partner** | **All aromatase inhibitors** | **2,214** | **460** | **811** | **0.554 (0.485-0.632)** | **<0.001** | **3.01** |
|  | Anastrozole | 999 | 211 | 345 | 0.612 (0.521-0.718) | <0.001 | 2.65 |
|  | Letrozole | 1,047 | 208 | 390 | 0.532 (0.454-0.623) | <0.001 | 3.17 |
|  | Exemestane | 168 | 41 | 76 | 0.547 (0.389-0.768) | <0.001 | 3.06 |
|  | **All non-AI regimens** | **554** | **129** | **238** | **0.669 (0.540-0.829)** | **<0.001** | **2.35** |
|  | Fulvestrant | 203 | 76 | 90 | 0.842 (0.652-1.088) | 0.187 | 1.66 |
|  | Tamoxifen | 351 | 53 | 148 | 0.360 (0.267-0.486) | <0.001 | 5 |
| **Metastatic burden** | ≥2 metastatic sites | 1,489 | 203 | 334 | 0.831 (0.698-0.989) | 0.032 | 1.7 |
|  | Visceral metastases | 1,710 | 222 | 385 | 0.814 (0.690-0.961) | 0.012 | 1.76 |
|  |  |  |  |  |  |  |  |

**Abbreviations**: AI, aromatase inhibitor; CI, confidence interval; HR, hazard ratio; AEs, adverse events.

**Data Source**: Analyses based on a propensity score-matched cohort (n=2,768 per group) to balance baseline characteristics.

**Subgroup Definitions**:

- **Age**: <65 vs. ≥65 years, based on CDK4/6 inhibitor initiation (median age cutpoint).
- **Menopausal Status**: Age ≥55 years, surgical menopause, or clinical documentation; undocumented patients excluded from this subgroup.
- **Sex**: Female or male, per electronic health record.
- **Race**: White, Asian, or Black/African American; unknown race (n=249 abemaciclib, n=242 palbociclib) excluded from race-specific analyses.
- **Brain Metastases**: Radiologically confirmed metastatic lesions in brain parenchyma/meninges at/before CDK4/6 inhibitor initiation.
- **Endocrine Partner**: Grouped as AI (anastrozole, letrozole, exemestane; n=2,214, 80.0%) vs. non-AI (fulvestrant, tamoxifen; n=554, 20.0%).
- **Metastatic Burden**:
  - ≥3 metastatic sites: ≥3 organ systems involved (n=1,489 per group, separately matched).
  - Visceral metastases: Confirmed involvement of visceral organs (lung, liver, bone marrow, peritoneal etc).

**Statistical Methods**:

- **HRs**: From stratified Cox models; HR <1.0 favors abemaciclib.
- **P-values**: Two-sided Wald test (α=0.05), unadjusted for multiple comparisons; subgroup analyses exploratory.
- **E-values**: Minimum confounder strength needed to explain HR; E-values >2.0 suggest robustness.
- **Individual Endocrine Analyses**: Exploratory, interpreted cautiously due to smaller sample sizes.
- **Interaction Tests**: No significant treatment effect heterogeneity across subgroups (P >0.05).

Note: *Statistically significant (95% CI excludes 1.0).

**eTable 10. Treatment Patterns and CDK4/6 Inhibitor Usage**

| **Characteristic** | **Abemaciclib (n=2,768)** | **Palbociclib (n=2,768)** |
| --- | --- | --- |
| Median time from diagnosis to CDK4/6i initiation (days) | 59 | 58 |
| **CDK4/6i treatment episodes** |  |  |
| Mean ± SD | 7.1 ± 9.6 | 7.7 ± 11.6 |
| Median | 4 | 4 |
|  |  |  |
| **Abbreviations:** SD, standard deviation; CDK4/6i, cyclin-dependent kinase 4/6 inhibitor. |  |  |

**eTable 11. Follow-up Duration and Overall Survival**

| **Characteristic** | **Before PSM** |  | **After PSM** |  |  |  |  |  |
| --- | --- | --- | --- | --- | --- | --- | --- | --- |
|  | Abemaciclib (n=2,808) | Palbociclib (n=8,904) | Abemaciclib (n=2,768) | Palbociclib (n=2,768) |  |  |  |  |
| **Follow-up duration** |  |  |  |  |  |  |  |  |
| Mean ± SD (days) | 1144.2 ± 527.1 | 1531.0 ± 802.9 | 1145.3 ± 528.2 | 1536.0 ± 808.7 |  |  |  |  |
| Median (IQR) (days) | 1027 (694) | 1344 (1143) | 1027 (695) | 1347 (1161) |  |  |  |  |
| **Overall survival** |  |  |  |  |  |  |  |  |
| Median OS (days) | 1863 | 1543 | 2206 | 1820 |  |  |  |  |
| Median OS (months) | 62.1 | 51.4 | 73.5 | 60.7 |  |  |  |  |
|  |  |  |  |  |  |  |  |  |
| **Abbreviations:** PSM, propensity score matching; SD, standard deviation; IQR, interquartile range; OS, overall survival. | | | | |  |  |  |  |

- eFigure 1. Kaplan−Meier Curves with Restricted Mean Survival Time (RMST) for overall survival (OS)

eFigure 2. Overall survival plots of sensitivity tests (On-Treatment, and **Landmark analysis)**

| **On-Treatment design: HR:0.79, 95%CI: 0.63 – 0.98** |
| --- |
| 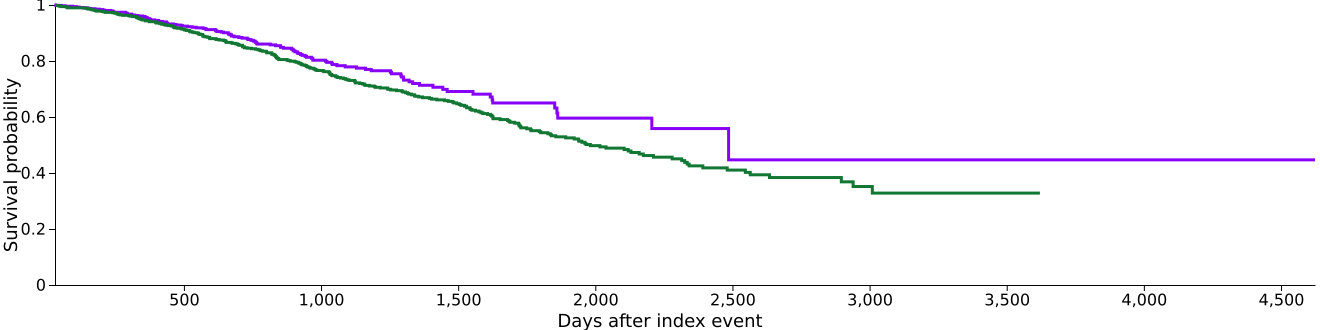 |
| **Landmark Analysis: HR:0.78, 95% CI : 0.69 – 0.87** |
| 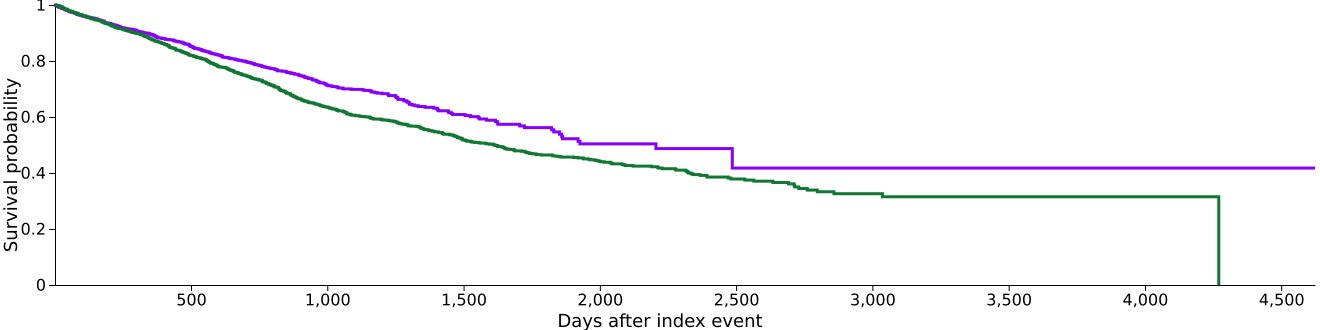 |
| **Calendar time–restricted analysis: HR:0.89, 95% CI : 0.80 – 0.99** |
| 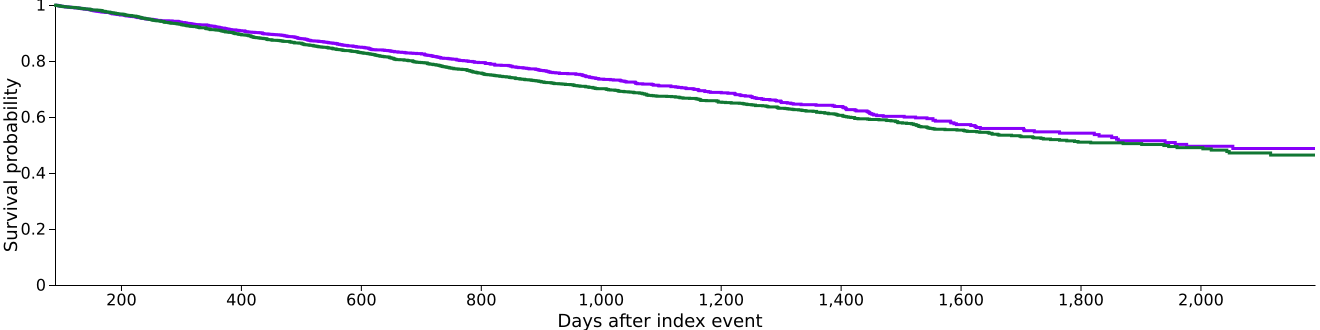 |

- **eFigure 3. − Propensity score density function of main cohort- Before and after matching (Abemaciclib - purple, Palbociclib - green)**

|  |  | 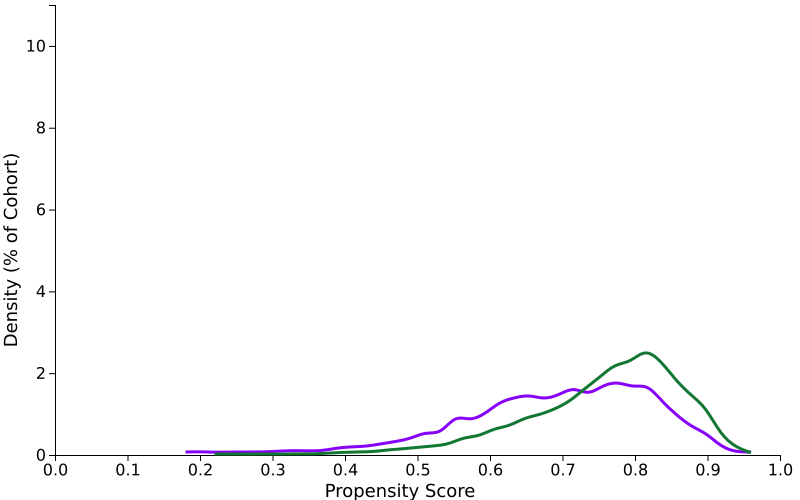 | 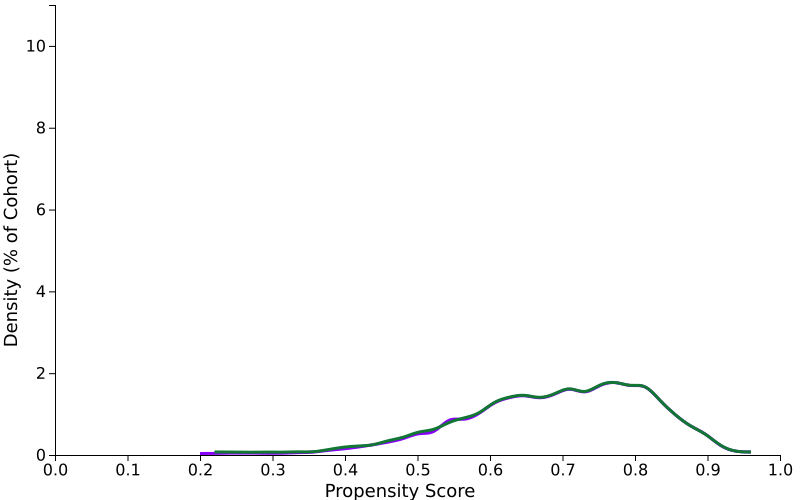 |
| --- | --- | --- | --- |

## eMethods. Additional Methodological Details

**Data Source:** We used the TriNetX Research Network, which includes de-identified electronic health record (EHR) data from more than 100 healthcare organizations globally. The network includes longitudinal data on demographics, diagnoses, procedures, medications, laboratory results, and clinical notes. Oncology-specific modules and medication records were utilized for this study, allowing comprehensive capture of breast cancer treatments and outcomes.

**Study Design:** We conducted a target trial emulation comparing abemaciclib vs palbociclib as first-line therapy for HR+/HER2- metastatic breast cancer. Two analytic strategies were applied:

1. As-started (primary): Follow patients from treatment initiation to death or censoring regardless of treatment changes (equivalent to intention-to-treat in randomized trials)
2. On-treatment (secondary): Follow patients from initiation until discontinuation of the index drug (≥6 consecutive doses required to confirm continuity)

**Propensity Score Matching:** Matching was conducted using a 1:1 nearest-neighbor algorithm with a caliper width of a 0.2 standard deviation of the logit of the propensity score. Variables adjusted for included: age, sex, race, BMI, ECOG performance status, comorbidities (hypertension, diabetes, cardiovascular disease, etc.), metastatic sites, prior medication use, and endocrine therapy partner (aromatase inhibitor, fulvestrant, tamoxifen). We assessed match quality using standardized mean differences, with values <0.1 indicating adequate balance.

**Outcomes:** The primary outcome was overall survival (OS), defined as time from treatment initiation to death from any cause. Secondary outcomes included hospitalization, ICU admission, severe infection, and adverse events categorized as hematologic (neutropenia, anemia, etc.) and non-hematologic (gastrointestinal, dermatologic, cardiac, etc.). Outcomes were captured using ICD-10 and CPT codes (detailed in eTable 2).

**Statistical Analyses:** Kaplan-Meier methods were used for time-to-event analyses, with survival curves compared using log-rank tests. Hazard ratios (HRs) and 95% confidence intervals (CIs) were estimated using Cox proportional hazards models with robust standard errors to account for matching. For binary outcomes, we calculated risk ratios at specific time points and over the entire follow-up period. Subgroup analyses were pre-specified for key patient characteristics (age, sex, race, metastatic pattern, endocrine partner), with interaction tests to assess heterogeneity of treatment effects.

**Sensitivity Analyses:** Multiple sensitivity analyses were conducted to test robustness of findings:

1. On-treatment analysis to assess efficacy while actively on medication
2. Analysis excluding outcomes within first month post-initiation to minimize immortal time bias
3. Negative control outcomes analysis to assess potential unmeasured confounding

**Software and Compliance:** All analyses were performed using R version 4.2 and the TriNetX analytics platform. This retrospective study was conducted in accordance with the Declaration of Helsinki and institutional data use agreements. The study was reviewed and approved by the institutional review board of the participating institutions with a waiver of informed consent for use of de-identified data.

**Abbreviations:** HR, hazard ratio; RR, risk ratio; OS, overall survival; ICU, intensive care unit; PSM, propensity score matching; AE, adverse event; RWD, real-world data; ICD-10, International Classification of Diseases 10th revision; CPT, Current Procedural Terminology.
